# Measurement of the $$\mathrm{t}\overline{{\mathrm{t}}}$$ production cross section in the all-jets final state in pp collisions at $$\sqrt{s}=8$$$$\,\text {TeV}$$

**DOI:** 10.1140/epjc/s10052-016-3956-5

**Published:** 2016-03-08

**Authors:** V. Khachatryan, A. M. Sirunyan, A. Tumasyan, W. Adam, E. Asilar, T. Bergauer, J. Brandstetter, E. Brondolin, M. Dragicevic, J. Erö, M. Friedl, R. Frühwirth, V. M. Ghete, C. Hartl, N. Hörmann, J. Hrubec, M. Jeitler, V. Knünz, A. König, M. Krammer, I. Krätschmer, D. Liko, T. Matsushita, I. Mikulec, D. Rabady, B. Rahbaran, H. Rohringer, J. Schieck, R. Schöfbeck, J. Strauss, W. Treberer-Treberspurg, W. Waltenberger, C.-E. Wulz, V. Mossolov, N. Shumeiko, J. Suarez Gonzalez, S. Alderweireldt, T. Cornelis, E. A. De Wolf, X. Janssen, A. Knutsson, J. Lauwers, S. Luyckx, M. Van De Klundert, H. Van Haevermaet, P. Van Mechelen, N. Van Remortel, A. Van Spilbeeck, S. Abu Zeid, F. Blekman, J. D’Hondt, N. Daci, I. De Bruyn, K. Deroover, N. Heracleous, J. Keaveney, S. Lowette, L. Moreels, A. Olbrechts, Q. Python, D. Strom, S. Tavernier, W. Van Doninck, P. Van Mulders, G. P. Van Onsem, I. Van Parijs, P. Barria, H. Brun, C. Caillol, B. Clerbaux, G. De Lentdecker, G. Fasanella, L. Favart, A. Grebenyuk, G. Karapostoli, T. Lenzi, A. Léonard, T. Maerschalk, A. Marinov, L. Perniè, A. Randle-conde, T. Reis, T. Seva, C. Vander Velde, R. Yonamine, P. Vanlaer, R. Yonamine, F. Zenoni, F. Zhang, V. Adler, K. Beernaert, L. Benucci, A. Cimmino, S. Crucy, D. Dobur, A. Fagot, G. Garcia, M. Gul, J. Mccartin, A. A. Ocampo Rios, D. Poyraz, D. Ryckbosch, S. Salva, M. Sigamani, N. Strobbe, M. Tytgat, W. Van Driessche, E. Yazgan, N. Zaganidis, S. Basegmez, C. Beluffi, O. Bondu, S. Brochet, G. Bruno, A. Caudron, L. Ceard, G. G. Da Silveira, C. Delaere, D. Favart, L. Forthomme, A. Giammanco, J. Hollar, A. Jafari, P. Jez, M. Komm, V. Lemaitre, A. Mertens, C. Nuttens, L. Perrini, A. Pin, K. Piotrzkowski, A. Popov, L. Quertenmont, M. Selvaggi, M. Vidal Marono, N. Beliy, G. H. Hammad, W. L. Aldá Júnior, F. L. Alves, G. A. Alves, L. Brito, M. Correa Martins Junior, M. Hamer, C. Hensel, C. Mora Herrera, A. Moraes, M. E. Pol, P. Rebello Teles, E. Belchior Batista Das Chagas, W. Carvalho, J. Chinellato, A. Custódio, E. M. Da Costa, D. De Jesus Damiao, C. De Oliveira Martins, S. Fonseca De Souza, L. M. Huertas Guativa, H. Malbouisson, D. Matos Figueiredo, L. Mundim, H. Nogima, W. L. Prado Da Silva, A. Santoro, A. Sznajder, E. J. Tonelli Manganote, A. Vilela Pereira, S. Ahuja, C. A. Bernardes, A. De Souza Santos, S. Dogra, T. R. Fernandez Perez Tomei, E. M. Gregores, P. G. Mercadante, C. S. Moon, S. F. Novaes, Sandra S. Padula, D. Romero Abad, J. C. Ruiz Vargas, A. Aleksandrov, R. Hadjiiska, P. Iaydjiev, M. Rodozov, S. Stoykova, G. Sultanov, M. Vutova, A. Dimitrov, I. Glushkov, L. Litov, B. Pavlov, P. Petkov, M. Ahmad, J. G. Bian, G. M. Chen, H. S. Chen, M. Chen, T. Cheng, R. Du, C. H. Jiang, R. Plestina, F. Romeo, S. M. Shaheen, J. Tao, C. Wang, Z. Wang, H. Zhang, C. Asawatangtrakuldee, Y. Ban, Q. Li, S. Liu, Y. Mao, S. J. Qian, D. Wang, Z. Xu, C. Avila, A. Cabrera, L. F. Chaparro Sierra, C. Florez, J. P. Gomez, B. Gomez Moreno, J. C. Sanabria, N. Godinovic, D. Lelas, I. Puljak, P. M. Ribeiro Cipriano, Z. Antunovic, M. Kovac, V. Brigljevic, K. Kadija, J. Luetic, S. Micanovic, L. Sudic, A. Attikis, G. Mavromanolakis, J. Mousa, C. Nicolaou, F. Ptochos, P. A. Razis, H. Rykaczewski, M. Bodlak, M. Finger, M. Finger, A. A. Abdelalim, A. Awad, M. El Sawy, A. Mahrous, A. Radi, B. Calpas, M. Kadastik, M. Murumaa, M. Raidal, A. Tiko, C. Veelken, P. Eerola, J. Pekkanen, M. Voutilainen, J. Härkönen, V. Karimäki, R. Kinnunen, T. Lampén, K. Lassila-Perini, S. Lehti, T. Lindén, P. Luukka, T. Mäenpää, T. Peltola, E. Tuominen, J. Tuominiemi, E. Tuovinen, L. Wendland, J. Talvitie, T. Tuuva, M. Besancon, F. Couderc, M. Dejardin, D. Denegri, B. Fabbro, J. L. Faure, C. Favaro, F. Ferri, S. Ganjour, A. Givernaud, P. Gras, G. Hamel de Monchenault, P. Jarry, E. Locci, M. Machet, J. Malcles, J. Rander, A. Rosowsky, M. Titov, A. Zghiche, I. Antropov, S. Baffioni, F. Beaudette, P. Busson, L. Cadamuro, E. Chapon, C. Charlot, T. Dahms, O. Davignon, N. Filipovic, A. Florent, R. Granier de Cassagnac, S. Lisniak, L. Mastrolorenzo, P. Miné, I. N. Naranjo, M. Nguyen, C. Ochando, G. Ortona, P. Paganini, P. Pigard, S. Regnard, R. Salerno, J. B. Sauvan, Y. Sirois, T. Strebler, Y. Yilmaz, A. Zabi, J.-L. Agram, J. Andrea, A. Aubin, D. Bloch, J.-M. Brom, M. Buttignol, E. C. Chabert, N. Chanon, C. Collard, E. Conte, X. Coubez, J.-C. Fontaine, D. Gelé, U. Goerlach, C. Goetzmann, A.-C. Le Bihan, J. A. Merlin, K. Skovpen, P. Van Hove, S. Gadrat, S. Beauceron, C. Bernet, G. Boudoul, E. Bouvier, C. A. Carrillo Montoya, R. Chierici, D. Contardo, B. Courbon, P. Depasse, H. El Mamouni, J. Fan, J. Fay, S. Gascon, M. Gouzevitch, B. Ille, F. Lagarde, I. B. Laktineh, M. Lethuillier, L. Mirabito, A. L. Pequegnot, S. Perries, J. D. Ruiz Alvarez, D. Sabes, L. Sgandurra, V. Sordini, M. Vander Donckt, P. Verdier, S. Viret, T. Toriashvili, Z. Tsamalaidze, C. Autermann, S. Beranek, M. Edelhoff, L. Feld, A. Heister, M. K. Kiesel, K. Klein, M. Lipinski, A. Ostapchuk, M. Preuten, F. Raupach, S. Schael, J. F. Schulte, T. Verlage, H. Weber, B. Wittmer, V. Zhukov, M. Ata, M. Brodski, E. Dietz-Laursonn, D. Duchardt, M. Endres, M. Erdmann, S. Erdweg, T. Esch, R. Fischer, A. Güth, T. Hebbeker, C. Heidemann, K. Hoepfner, D. Klingebiel, S. Knutzen, P. Kreuzer, M. Merschmeyer, A. Meyer, P. Millet, M. Olschewski, K. Padeken, P. Papacz, T. Pook, M. Radziej, H. Reithler, M. Rieger, F. Scheuch, L. Sonnenschein, D. Teyssier, S. Thüer, V. Cherepanov, Y. Erdogan, G. Flügge, H. Geenen, M. Geisler, F. Hoehle, B. Kargoll, T. Kress, Y. Kuessel, A. Künsken, J. Lingemann, A. Nehrkorn, A. Nowack, I. M. Nugent, C. Pistone, O. Pooth, A. Stahl, M. Aldaya Martin, I. Asin, N. Bartosik, O. Behnke, U. Behrens, A. J. Bell, K. Borras, A. Burgmeier, A. Cakir, L. Calligaris, A. Campbell, S. Choudhury, F. Costanza, C. Diez Pardos, G. Dolinska, S. Dooling, T. Dorland, G. Eckerlin, D. Eckstein, T. Eichhorn, G. Flucke, E. Gallo, J. Garay Garcia, A. Geiser, A. Gizhko, P. Gunnellini, J. Hauk, M. Hempel, H. Jung, A. Kalogeropoulos, O. Karacheban, M. Kasemann, P. Katsas, J. Kieseler, C. Kleinwort, I. Korol, W. Lange, J. Leonard, K. Lipka, A. Lobanov, W. Lohmann, R. Mankel, I. Marfin, I.-A. Melzer-Pellmann, A. B. Meyer, G. Mittag, J. Mnich, A. Mussgiller, S. Naumann-Emme, A. Nayak, E. Ntomari, H. Perrey, D. Pitzl, R. Placakyte, A. Raspereza, B. Roland, M. Ö. Sahin, P. Saxena, T. Schoerner-Sadenius, M. Schröder, C. Seitz, S. Spannagel, K. D. Trippkewitz, R. Walsh, C. Wissing, V. Blobel, M. Centis Vignali, A. R. Draeger, J. Erfle, E. Garutti, K. Goebel, D. Gonzalez, M. Görner, J. Haller, M. Hoffmann, R. S. Höing, A. Junkes, R. Klanner, R. Kogler, T. Lapsien, T. Lenz, I. Marchesini, D. Marconi, M. Meyer, D. Nowatschin, J. Ott, F. Pantaleo, T. Peiffer, A. Perieanu, N. Pietsch, J. Poehlsen, D. Rathjens, C. Sander, H. Schettler, P. Schleper, E. Schlieckau, A. Schmidt, J. Schwandt, M. Seidel, V. Sola, H. Stadie, G. Steinbrück, H. Tholen, D. Troendle, E. Usai, L. Vanelderen, A. Vanhoefer, B. Vormwald, M. Akbiyik, C. Barth, C. Baus, J. Berger, C. Böser, E. Butz, T. Chwalek, F. Colombo, W. De Boer, A. Descroix, A. Dierlamm, S. Fink, F. Frensch, M. Giffels, A. Gilbert, F. Hartmann, S. M. Heindl, U. Husemann, I. Katkov, A. Kornmayer, P. Lobelle Pardo, B. Maier, H. Mildner, M. U. Mozer, T. Müller, Th. Müller, M. Plagge, G. Quast, K. Rabbertz, S. Röcker, F. Roscher, H. J. Simonis, F. M. Stober, R. Ulrich, J. Wagner-Kuhr, S. Wayand, M. Weber, T. Weiler, C. Wöhrmann, R. Wolf, G. Anagnostou, G. Daskalakis, T. Geralis, V. A. Giakoumopoulou, A. Kyriakis, D. Loukas, A. Psallidas, I. Topsis-Giotis, A. Agapitos, S. Kesisoglou, A. Panagiotou, N. Saoulidou, E. Tziaferi, I. Evangelou, G. Flouris, C. Foudas, P. Kokkas, N. Loukas, N. Manthos, I. Papadopoulos, E. Paradas, J. Strologas, G. Bencze, C. Hajdu, A. Hazi, P. Hidas, D. Horvath, F. Sikler, V. Veszpremi, G. Vesztergombi, A. J. Zsigmond, N. Beni, S. Czellar, J. Karancsi, J. Molnar, Z. Szillasi, M. Bartók, A. Makovec, P. Raics, Z. L. Trocsanyi, B. Ujvari, P. Mal, K. Mandal, D. K. Sahoo, N. Sahoo, S. K. Swain, S. Bansal, S. B. Beri, V. Bhatnagar, R. Chawla, R. Gupta, U. Bhawandeep, A. K. Kalsi, A. Kaur, M. Kaur, R. Kumar, A. Mehta, M. Mittal, J. B. Singh, G. Walia, Ashok Kumar, A. Bhardwaj, B. C. Choudhary, R. B. Garg, A. Kumar, S. Malhotra, M. Naimuddin, N. Nishu, K. Ranjan, R. Sharma, V. Sharma, S. Bhattacharya, K. Chatterjee, S. Dey, S. Dutta, Sa. Jain, N. Majumdar, A. Modak, K. Mondal, S. Mukherjee, S. Mukhopadhyay, A. Roy, D. Roy, S. Roy Chowdhury, S. Sarkar, M. Sharan, A. Abdulsalam, R. Chudasama, D. Dutta, V. Jha, V. Kumar, A. K. Mohanty, L. M. Pant, P. Shukla, A. Topkar, T. Aziz, S. Banerjee, S. Bhowmik, R. M. Chatterjee, R. K. Dewanjee, S. Dugad, S. Ganguly, S. Ghosh, M. Guchait, A. Gurtu, G. Kole, S. Kumar, B. Mahakud, M. Maity, G. Majumder, K. Mazumdar, S. Mitra, G. B. Mohanty, B. Parida, T. Sarkar, N. Sur, B. Sutar, N. Wickramage, S. Chauhan, S. Dube, S. Sharma, H. Bakhshiansohi, H. Behnamian, S. M. Etesami, A. Fahim, R. Goldouzian, M. Khakzad, M. Mohammadi Najafabadi, M. Naseri, S. Paktinat Mehdiabadi, F. Rezaei Hosseinabadi, B. Safarzadeh, M. Zeinali, M. Felcini, M. Grunewald, M. Abbrescia, C. Calabria, C. Caputo, A. Colaleo, D. Creanza, L. Cristella, N. De Filippis, M. De Palma, L. Fiore, G. Iaselli, G. Maggi, G. Miniello, M. Maggi, S. My, S. Nuzzo, A. Pompili, G. Pugliese, R. Radogna, A. Ranieri, G. Selvaggi, L. Silvestris, R. Venditti, P. Verwilligen, G. Abbiendi, C. Battilana, A. C. Benvenuti, D. Bonacorsi, S. Braibant-Giacomelli, L. Brigliadori, R. Campanini, P. Capiluppi, A. Castro, F. R. Cavallo, S. S. Chhibra, G. Codispoti, M. Cuffiani, G. M. Dallavalle, F. Fabbri, A. Fanfani, D. Fasanella, P. Giacomelli, C. Grandi, L. Guiducci, S. Marcellini, G. Masetti, A. Montanari, F. L. Navarria, A. Perrotta, A. M. Rossi, F. Primavera, T. Rovelli, G. P. Siroli, N. Tosi, R. Travaglini, G. Cappello, M. Chiorboli, S. Costa, F. Giordano, R. Potenza, A. Tricomi, C. Tuve, G. Barbagli, V. Ciulli, C. Civinini, R. D’Alessandro, E. Focardi, S. Gonzi, V. Gori, P. Lenzi, M. Meschini, S. Paoletti, G. Sguazzoni, A. Tropiano, L. Viliani, L. Benussi, S. Bianco, F. Fabbri, D. Piccolo, F. Primavera, V. Calvelli, F. Ferro, M. Lo Vetere, M. R. Monge, E. Robutti, S. Tosi, L. Brianza, M. E. Dinardo, S. Fiorendi, S. Gennai, R. Gerosa, A. Ghezzi, P. Govoni, S. Malvezzi, R. A. Manzoni, B. Marzocchi, D. Menasce, L. Moroni, M. Paganoni, D. Pedrini, S. Ragazzi, N. Redaelli, T. Tabarelli de Fatis, S. Buontempo, N. Cavallo, S. Di Guida, M. Esposito, F. Fabozzi, A. O. M. Iorio, G. Lanza, L. Lista, S. Meola, M. Merola, P. Paolucci, C. Sciacca, F. Thyssen, P. Azzi, N. Bacchetta, L. Benato, D. Bisello, A. Boletti, R. Carlin, P. Checchia, M. Dall’Osso, T. Dorigo, F. Gasparini, U. Gasparini, A. Gozzelino, S. Lacaprara, M. Margoni, A. T. Meneguzzo, M. Michelotto, F. Montecassiano, M. Passaseo, J. Pazzini, M. Pegoraro, N. Pozzobon, P. Ronchese, F. Simonetto, E. Torassa, M. Tosi, S. Vanini, M. Zanetti, P. Zotto, A. Zucchetta, A. Braghieri, A. Magnani, P. Montagna, S. P. Ratti, V. Re, C. Riccardi, P. Salvini, I. Vai, P. Vitulo, L. Alunni Solestizi, M. Biasini, G. M. Bilei, D. Ciangottini, L. Fanò, P. Lariccia, G. Mantovani, M. Menichelli, A. Saha, A. Santocchia, A. Spiezia, K. Androsov, P. Azzurri, G. Bagliesi, J. Bernardini, T. Boccali, G. Broccolo, R. Castaldi, M. A. Ciocci, R. Dell’Orso, S. Donato, G. Fedi, F. Fiori, L. Foà, A. Giassi, M. T. Grippo, F. Ligabue, T. Lomtadze, L. Martini, A. Messineo, F. Palla, A. Rizzi, A. Savoy-Navarro, A. T. Serban, P. Spagnolo, P. Squillacioti, R. Tenchini, G. Tonelli, A. Venturi, P. G. Verdini, L. Barone, F. Cavallari, G. D’imperio, D. Del Re, M. Diemoz, S. Gelli, C. Jorda, E. Longo, F. Margaroli, P. Meridiani, G. Organtini, R. Paramatti, F. Preiato, S. Rahatlou, C. Rovelli, F. Santanastasio, P. Traczyk, N. Amapane, R. Arcidiacono, S. Argiro, M. Arneodo, R. Bellan, C. Biino, N. Cartiglia, M. Costa, R. Covarelli, A. Degano, N. Demaria, L. Finco, B. Kiani, C. Mariotti, S. Maselli, E. Migliore, V. Monaco, E. Monteil, M. Musich, M. M. Obertino, L. Pacher, N. Pastrone, M. Pelliccioni, G. L. Pinna Angioni, F. Ravera, A. Potenza, A. Romero, M. Ruspa, R. Sacchi, A. Solano, A. Staiano, U. Tamponi, S. Belforte, V. Candelise, M. Casarsa, F. Cossutti, G. Della Ricca, B. Gobbo, C. La Licata, M. Marone, A. Schizzi, A. Zanetti, T. A. Kropivnitskaya, S. K. Nam, D. H. Kim, G. N. Kim, M. S. Kim, M. S. Kim, D. J. Kong, S. Lee, Y. D. Oh, A. Sakharov, D. C. Son, J. A. Brochero Cifuentes, H. Kim, T. J. Kim, S. Song, S. Choi, Y. Go, D. Gyun, B. Hong, M. Jo, H. Kim, Y. Kim, B. Lee, K. Lee, K. S. Lee, S. Lee, S. Lee, S. K. Park, Y. Roh, H. D. Yoo, M. Choi, H. Kim, J. H. Kim, J. S. H. Lee, I. C. Park, G. Ryu, M. S. Ryu, Y. Choi, J. Goh, D. Kim, E. Kwon, J. Lee, I. Yu, A. Juodagalvis, J. Vaitkus, I. Ahmed, Z. A. Ibrahim, J. R. Komaragiri, M. A. B. Md Ali, F. Mohamad Idris, W. A. T. Wan Abdullah, M. N. Yusli, W. A. T. Wan Abdullah, E. Casimiro Linares, H. Castilla-Valdez, E. De La Cruz-Burelo, I. Heredia-De La Cruz, A. Hernandez-Almada, R. Lopez-Fernandez, A. Sanchez-Hernandez, S. Carrillo Moreno, F. Vazquez Valencia, I. Pedraza, H. A. Salazar Ibarguen, A. Morelos Pineda, D. Krofcheck, P. H. Butler, A. Ahmad, M. Ahmad, Q. Hassan, H. R. Hoorani, W. A. Khan, T. Khurshid, M. Shoaib, H. Bialkowska, M. Bluj, B. Boimska, T. Frueboes, M. Górski, M. Kazana, K. Nawrocki, K. Romanowska-Rybinska, M. Szleper, P. Zalewski, G. Brona, K. Bunkowski, A. Byszuk, K. Doroba, A. Kalinowski, M. Konecki, J. Krolikowski, M. Misiura, M. Olszewski, M. Walczak, P. Bargassa, C. Beir ao Da Cruz E Silva, A. Di Francesco, P. Faccioli, P. G. Ferreira Parracho, M. Gallinaro, N. Leonardo, L. Lloret Iglesias, F. Nguyen, J. Rodrigues Antunes, J. Seixas, O. Toldaiev, D. Vadruccio, J. Varela, P. Vischia, S. Afanasiev, P. Bunin, M. Gavrilenko, I. Golutvin, I. Gorbunov, A. Kamenev, V. Karjavin, V. Konoplyanikov, A. Lanev, A. Malakhov, V. Matveev, P. Moisenz, V. Palichik, V. Perelygin, M. Savina, S. Shmatov, S. Shulha, V. Smirnov, A. Zarubin, V. Golovtsov, Y. Ivanov, V. Kim, E. Kuznetsova, P. Levchenko, V. Murzin, V. Oreshkin, I. Smirnov, V. Sulimov, L. Uvarov, S. Vavilov, A. Vorobyev, Yu. Andreev, A. Dermenev, S. Gninenko, N. Golubev, A. Karneyeu, M. Kirsanov, N. Krasnikov, A. Pashenkov, D. Tlisov, A. Toropin, V. Epshteyn, V. Gavrilov, N. Lychkovskaya, V. Popov, l. Pozdnyakov, G. Safronov, A. Spiridonov, E. Vlasov, A. Zhokin, A. Bylinkin, V. Andreev, M. Azarkin, I. Dremin, M. Kirakosyan, A. Leonidov, G. Mesyats, S. V. Rusakov, A. Baskakov, A. Belyaev, E. Boos, V. Bunichev, M. Dubinin, L. Dudko, A. Ershov, A. Gribushin, V. Klyukhin, N. Korneeva, I. Lokhtin, I. Myagkov, S. Obraztsov, M. Perfilov, V. Savrin, I. Azhgirey, I. Bayshev, S. Bitioukov, V. Kachanov, A. Kalinin, D. Konstantinov, V. Krychkine, V. Petrov, R. Ryutin, A. Sobol, L. Tourtchanovitch, S. Troshin, N. Tyurin, A. Uzunian, A. Volkov, P. Adzic, J. Milosevic, V. Rekovic, J. Alcaraz Maestre, C. Battilana, E. Calvo, M. Cerrada, M. Chamizo Llatas, N. Colino, B. De La Cruz, A. Delgado Peris, D. Domínguez Vázquez, A. Escalante Del Valle, C. Fernandez Bedoya, J. P. Fernández Ramos, J. Flix, M. C. Fouz, P. Garcia-Abia, O. Gonzalez Lopez, S. Goy Lopez, J. M. Hernandez, M. I. Josa, E. Navarro De Martino, A. Pérez-Calero Yzquierdo, J. Puerta Pelayo, A. Quintario Olmeda, I. Redondo, L. Romero, J. Santaolalla, M. S. Soares, C. Albajar, J. F. de Trocóniz, M. Missiroli, D. Moran, J. Cuevas, J. Fernandez Menendez, S. Folgueras, I. Gonzalez Caballero, E. Palencia Cortezon, J. M. Vizan Garcia, I. J. Cabrillo, A. Calderon, J. R. Castiñeiras De Saa, P. De Castro Manzano, J. Duarte Campderros, M. Fernandez, J. Garcia-Ferrero, G. Gomez, A. Lopez Virto, J. Marco, R. Marco, C. Martinez Rivero, F. Matorras, F. J. Munoz Sanchez, J. Piedra Gomez, T. Rodrigo, A. Y. Rodríguez-Marrero, A. Ruiz-Jimeno, L. Scodellaro, N. Trevisani, I. Vila, R. Vilar Cortabitarte, D. Abbaneo, E. Auffray, G. Auzinger, M. Bachtis, P. Baillon, A. H. Ball, D. Barney, A. Benaglia, J. Bendavid, L. Benhabib, J. F. Benitez, G. M. Berruti, P. Bloch, A. Bocci, A. Bonato, C. Botta, H. Breuker, T. Camporesi, R. Castello, G. Cerminara, M. D’Alfonso, D. d’Enterria, A. Dabrowski, V. Daponte, A. David, M. De Gruttola, F. De Guio, A. De Roeck, S. De Visscher, E. Di Marco, M. Dobson, M. Dordevic, B. Dorney, T. du Pree, M. Dünser, N. Dupont, A. Elliott-Peisert, G. Franzoni, W. Funk, D. Gigi, K. Gill, D. Giordano, M. Girone, F. Glege, R. Guida, S. Gundacker, M. Guthoff, J. Hammer, P. Harris, J. Hegeman, V. Innocente, P. Janot, H. Kirschenmann, M. J. Kortelainen, K. Kousouris, K. Krajczar, P. Lecoq, C. Lourenço, M. T. Lucchini, N. Magini, L. Malgeri, M. Mannelli, A. Martelli, L. Masetti, F. Meijers, S. Mersi, E. Meschi, F. Moortgat, S. Morovic, M. Mulders, M. V. Nemallapudi, H. Neugebauer, S. Orfanelli, L. Orsini, L. Pape, E. Perez, M. Peruzzi, A. Petrilli, G. Petrucciani, A. Pfeiffer, D. Piparo, A. Racz, G. Rolandi, M. Rovere, M. Ruan, H. Sakulin, C. Schäfer, C. Schwick, A. Sharma, P. Silva, M. Simon, P. Sphicas, J. Steggemann, B. Stieger, M. Stoye, Y. Takahashi, D. Treille, A. Triossi, A. Tsirou, G. I. Veres, N. Wardle, H. K. Wöhri, A. Zagozdzinska, W. D. Zeuner, W. Bertl, K. Deiters, W. Erdmann, R. Horisberger, Q. Ingram, H. C. Kaestli, D. Kotlinski, U. Langenegger, D. Renker, T. Rohe, F. Bachmair, L. Bäni, L. Bianchini, B. Casal, G. Dissertori, M. Dittmar, M. Donegà, P. Eller, C. Grab, C. Heidegger, D. Hits, J. Hoss, G. Kasieczka, W. Lustermann, B. Mangano, M. Marionneau, P. Martinez Ruiz del Arbol, M. Masciovecchio, D. Meister, F. Micheli, P. Musella, F. Nessi-Tedaldi, F. Pandolfi, J. Pata, F. Pauss, L. Perrozzi, M. Quittnat, M. Rossini, A. Starodumov, M. Takahashi, V. R. Tavolaro, K. Theofilatos, R. Wallny, T. K. Aarrestad, C. Amsler, L. Caminada, M. F. Canelli, V. Chiochia, A. De Cosa, C. Galloni, A. Hinzmann, T. Hreus, B. Kilminster, C. Lange, J. Ngadiuba, D. Pinna, P. Robmann, F. J. Ronga, D. Salerno, Y. Yang, M. Cardaci, K. H. Chen, T. H. Doan, Sh. Jain, R. Khurana, M. Konyushikhin, C. M. Kuo, W. Lin, Y. J. Lu, S. S. Yu, Arun Kumar, R. Bartek, P. Chang, Y. H. Chang, Y. Chao, K. F. Chen, P. H. Chen, C. Dietz, F. Fiori, U. Grundler, W.-S. Hou, Y. Hsiung, Y. F. Liu, R.-S. Lu, M. Miñano Moya, E. Petrakou, J. f. Tsai, Y. M. Tzeng, B. Asavapibhop, K. Kovitanggoon, G. Singh, N. Srimanobhas, N. Suwonjandee, A. Adiguzel, M. N. Bakirci, Z. S. Demiroglu, C. Dozen, I. Dumanoglu, E. Eskut, S. Girgis, G. Gokbulut, Y. Guler, Y. Guler, E. Gurpinar, I. Hos, E. E. Kangal, A. Kayis Topaksu, G. Onengut, K. Ozdemir, A. Polatoz, D. Sunar Cerci, M. Vergili, C. Zorbilmez, I. V. Akin, B. Bilin, S. Bilmis, B. Isildak, G. Karapinar, M. Yalvac, M. Zeyrek, A. Albayrak, E. Gülmez, M. Kaya, O. Kaya, T. Yetkin, K. Cankocak, S. Sen, F. I. Vardarlı, B. Grynyov, L. Levchuk, P. Sorokin, R. Aggleton, F. Ball, L. Beck, J. J. Brooke, E. Clement, D. Cussans, H. Flacher, J. Goldstein, M. Grimes, G. P. Heath, H. F. Heath, J. Jacob, L. Kreczko, C. Lucas, Z. Meng, D. M. Newbold, S. Paramesvaran, A. Poll, T. Sakuma, S. Seif El Nasr-storey, S. Senkin, D. Smith, V. J. Smith, K. W. Bell, A. Belyaev, C. Brew, R. M. Brown, D. Cieri, D. J. A. Cockerill, J. A. Coughlan, K. Harder, S. Harper, E. Olaiya, D. Petyt, C. H. Shepherd-Themistocleous, A. Thea, I. R. Tomalin, T. Williams, W. J. Womersley, S. D. Worm, M. Baber, R. Bainbridge, O. Buchmuller, A. Bundock, D. Burton, S. Casasso, M. Citron, D. Colling, L. Corpe, N. Cripps, P. Dauncey, G. Davies, A. De Wit, M. Della Negra, P. Dunne, A. Elwood, A. Elwood, W. Ferguson, J. Fulcher, D. Futyan, G. Hall, G. Iles, M. Kenzie, R. Lane, R. Lucas, L. Lyons, A.-M. Magnan, S. Malik, J. Nash, A. Nikitenko, J. Pela, M. Pesaresi, K. Petridis, D. M. Raymond, A. Richards, A. Rose, C. Seez, A. Tapper, K. Uchida, M. Vazquez Acosta, T. Virdee, S. C. Zenz, J. E. Cole, P. R. Hobson, A. Khan, P. Kyberd, D. Leggat, D. Leslie, I. D. Reid, P. Symonds, L. Teodorescu, M. Turner, A. Borzou, K. Call, J. Dittmann, K. Hatakeyama, A. Kasmi, H. Liu, N. Pastika, T. Scarborough, Z. Wu, O. Charaf, S. I. Cooper, C. Henderson, P. Rumerio, A. Avetisyan, T. Bose, C. Fantasia, D. Gastler, P. Lawson, D. Rankin, C. Richardson, J. Rohlf, J. St. John, L. Sulak, D. Zou, J. Alimena, E. Berry, S. Bhattacharya, D. Cutts, N. Dhingra, A. Ferapontov, A. Garabedian, J. Hakala, U. Heintz, E. Laird, G. Landsberg, Z. Mao, M. Narain, S. Piperov, S. Sagir, T. Sinthuprasith, R. Syarif, R. Breedon, G. Breto, M. Calderon De La Barca Sanchez, S. Chauhan, M. Chertok, J. Conway, R. Conway, P. T. Cox, R. Erbacher, M. Gardner, W. Ko, R. Lander, M. Mulhearn, D. Pellett, J. Pilot, F. Ricci-Tam, S. Shalhout, J. Smith, M. Squires, D. Stolp, M. Tripathi, S. Wilbur, R. Yohay, R. Cousins, P. Everaerts, C. Farrell, J. Hauser, M. Ignatenko, D. Saltzberg, V. Valuev, M. Weber, K. Burt, R. Clare, J. Ellison, J. W. Gary, G. Hanson, J. Heilman, M. Ivova PANEVA, P. Jandir, E. Kennedy, F. Lacroix, O. R. Long, A. Luthra, M. Malberti, M. Olmedo Negrete, A. Shrinivas, H. Wei, S. Wimpenny, B. R. Yates, J. G. Branson, G. B. Cerati, S. Cittolin, R. T. D’Agnolo, A. Holzner, R. Kelley, D. Klein, J. Letts, I. Macneill, D. Olivito, S. Padhi, M. Pieri, M. Sani, V. Sharma, S. Simon, M. Tadel, Y. Tu, A. Vartak, S. Wasserbaech, C. Welke, F. Würthwein, A. Yagil, G. Zevi Della Porta, D. Barge, J. Bradmiller-Feld, C. Campagnari, A. Dishaw, V. Dutta, K. Flowers, M. Franco Sevilla, P. Geffert, C. George, F. Golf, L. Gouskos, J. Gran, J. Incandela, C. Justus, N. Mccoll, S. D. Mullin, S. D. Mullin, J. Richman, D. Stuart, I. Suarez, W. To, C. West, J. Yoo, D. Anderson, A. Apresyan, A. Bornheim, J. Bunn, Y. Chen, J. Duarte, A. Mott, H. B. Newman, C. Pena, M. Pierini, M. Spiropulu, J. R. Vlimant, S. Xie, R. Y. Zhu, M. B. Andrews, V. Azzolini, A. Calamba, B. Carlson, T. Ferguson, M. Paulini, J. Russ, M. Sun, H. Vogel, I. Vorobiev, J. P. Cumalat, W. T. Ford, A. Gaz, F. Jensen, A. Johnson, M. Krohn, T. Mulholland, U. Nauenberg, K. Stenson, S. R. Wagner, J. Alexander, A. Chatterjee, J. Chaves, J. Chu, S. Dittmer, N. Eggert, N. Mirman, G. Nicolas Kaufman, J. R. Patterson, A. Rinkevicius, A. Ryd, L. Skinnari, L. Soffi, W. Sun, S. M. Tan, W. D. Teo, J. Thom, J. Thompson, J. Tucker, Y. Weng, P. Wittich, S. Abdullin, M. Albrow, J. Anderson, G. Apollinari, S. Banerjee, L. A. T. Bauerdick, A. Beretvas, J. Berryhill, P. C. Bhat, G. Bolla, K. Burkett, J. N. Butler, H. W. K. Cheung, F. Chlebana, S. Cihangir, V. D. Elvira, I. Fisk, J. Freeman, E. Gottschalk, L. Gray, D. Green, S. Grünendahl, O. Gutsche, J. Hanlon, D. Hare, R. M. Harris, S. Hasegawa, J. Hirschauer, Z. Hu, S. Jindariani, M. Johnson, U. Joshi, A. W. Jung, B. Klima, B. Kreis, S. Kwan, S. Lammel, J. Linacre, D. Lincoln, R. Lipton, T. Liu, R. Lopes De Sá, J. Lykken, K. Maeshima, J. M. Marraffino, V. I. Martinez Outschoorn, S. Maruyama, D. Mason, P. McBride, P. Merkel, K. Mishra, S. Mrenna, S. Nahn, C. Newman-Holmes, V. O’Dell, K. Pedro, O. Prokofyev, G. Rakness, E. Sexton-Kennedy, A. Soha, W. J. Spalding, L. Spiegel, L. Taylor, S. Tkaczyk, N. V. Tran, L. Uplegger, E. W. Vaandering, C. Vernieri, M. Verzocchi, R. Vidal, H. A. Weber, A. Whitbeck, F. Yang, D. Acosta, P. Avery, P. Bortignon, D. Bourilkov, A. Carnes, M. Carver, D. Curry, S. Das, G. P. Di Giovanni, R. D. Field, I. K. Furic, J. Hugon, J. Konigsberg, A. Korytov, J. F. Low, P. Ma, K. Matchev, H. Mei, P. Milenovic, G. Mitselmakher, D. Rank, R. Rossin, L. Shchutska, M. Snowball, D. Sperka, N. Terentyev, L. Thomas, J. Wang, S. Wang, J. Yelton, S. Hewamanage, S. Linn, P. Markowitz, G. Martinez, J. L. Rodriguez, J. R. Adams, A. Ackert, T. Adams, A. Askew, J. Bochenek, B. Diamond, J. Haas, S. Hagopian, V. Hagopian, K. F. Johnson, A. Khatiwada, H. Prosper, M. Weinberg, M. M. Baarmand, V. Bhopatkar, S. Colafranceschi, M. Hohlmann, H. Kalakhety, D. Noonan, T. Roy, F. Yumiceva, M. R. Adams, L. Apanasevich, D. Berry, R. R. Betts, I. Bucinskaite, R. Cavanaugh, O. Evdokimov, L. Gauthier, C. E. Gerber, D. J. Hofman, P. Kurt, C. O’Brien, l. D. Sandoval Gonzalez, C. Silkworth, P. Turner, N. Varelas, Z. Wu, M. Zakaria, B. Bilki, W. Clarida, K. Dilsiz, S. Durgut, R. P. Gandrajula, M. Haytmyradov, V. Khristenko, J.-P. Merlo, H. Mermerkaya, A. Mestvirishvili, A. Moeller, J. Nachtman, H. Ogul, Y. Onel, F. Ozok, A. Penzo, C. Snyder, E. Tiras, J. Wetzel, K. Yi, I. Anderson, I. Anderson, B. A. Barnett, B. Blumenfeld, N. Eminizer, D. Fehling, L. Feng, A. V. Gritsan, P. Maksimovic, C. Martin, M. Osherson, J. Roskes, A. Sady, U. Sarica, M. Swartz, M. Xiao, Y. Xin, C. You, M. Xiao, P. Baringer, A. Bean, G. Benelli, C. Bruner, R. P. Kenny, D. Majumder, D. Majumder, M. Malek, M. Murray, S. Sanders, R. Stringer, Q. Wang, A. Ivanov, K. Kaadze, S. Khalil, M. Makouski, Y. Maravin, A. Mohammadi, L. K. Saini, N. Skhirtladze, S. Toda, D. Lange, F. Rebassoo, D. Wright, C. Anelli, A. Baden, O. Baron, A. Belloni, B. Calvert, S. C. Eno, C. Ferraioli, J. A. Gomez, N. J. Hadley, S. Jabeen, S. Jabeen, R. G. Kellogg, T. Kolberg, J. Kunkle, Y. Lu, A. C. Mignerey, Y. H. Shin, A. Skuja, M. B. Tonjes, S. C. Tonwar, A. Apyan, R. Barbieri, A. Baty, K. Bierwagen, S. Brandt, K. Bierwagen, W. Busza, I. A. Cali, Z. Demiragli, L. Di Matteo, G. Gomez Ceballos, M. Goncharov, D. Gulhan, Y. Iiyama, G. M. Innocenti, M. Klute, D. Kovalskyi, Y. S. Lai, Y.-J. Lee, A. Levin, P. D. Luckey, A. C. Marini, C. Mcginn, C. Mironov, X. Niu, C. Paus, D. Ralph, C. Roland, G. Roland, J. Salfeld-Nebgen, G. S. F. Stephans, K. Sumorok, M. Varma, D. Velicanu, J. Veverka, J. Wang, T. W. Wang, B. Wyslouch, M. Yang, V. Zhukova, B. Dahmes, A. Evans, A. Finkel, A. Gude, P. Hansen, S. Kalafut, S. C. Kao, K. Klapoetke, Y. Kubota, Z. Lesko, J. Mans, S. Nourbakhsh, N. Ruckstuhl, R. Rusack, N. Tambe, J. Turkewitz, J. G. Acosta, S. Oliveros, E. Avdeeva, K. Bloom, S. Bose, D. R. Claes, A. Dominguez, C. Fangmeier, R. Gonzalez Suarez, R. Kamalieddin, J. Keller, D. Knowlton, I. Kravchenko, J. Lazo-Flores, F. Meier, J. Monroy, F. Ratnikov, J. E. Siado, G. R. Snow, M. Alyari, J. Dolen, J. George, A. Godshalk, C. Harrington, I. Iashvili, J. Kaisen, A. Kharchilava, A. Kumar, S. Rappoccio, B. Roozbahani, G. Alverson, E. Barberis, D. Baumgartel, M. Chasco, A. Hortiangtham, A. Massironi, D. M. Morse, D. Nash, T. Orimoto, R. Teixeira De Lima, D. Trocino, R.-J. Wang, D. Wood, J. Zhang, K. A. Hahn, A. Kubik, N. Mucia, N. Odell, B. Pollack, A. Pozdnyakov, M. Schmitt, S. Stoynev, K. Sung, M. Trovato, M. Velasco, A. Brinkerhoff, N. Dev, M. Hildreth, C. Jessop, D. J. Karmgard, N. Kellams, K. Lannon, S. Lynch, N. Marinelli, F. Meng, C. Mueller, Y. Musienko, T. Pearson, M. Planer, A. Reinsvold, R. Ruchti, G. Smith, S. Taroni, N. Valls, M. Wayne, M. Wolf, A. Woodard, L. Antonelli, J. Brinson, B. Bylsma, L. S. Durkin, S. Flowers, A. Hart, C. Hill, R. Hughes, W. Ji, K. Kotov, T. Y. Ling, B. Liu, W. Luo, D. Puigh, M. Rodenburg, B. L. Winer, H. W. Wulsin, O. Driga, P. Elmer, J. Hardenbrook, P. Hebda, S. A. Koay, P. Lujan, D. Marlow, T. Medvedeva, M. Mooney, J. Olsen, C. Palmer, P. Piroué, X. Quan, H. Saka, D. Stickland, C. Tully, J. S. Werner, A. Zuranski, S. Malik, V. E. Barnes, D. Benedetti, D. Bortoletto, L. Gutay, M. K. Jha, M. Jones, K. Jung, D. H. Miller, N. Neumeister, F. Primavera, B. C. Radburn-Smith, X. Shi, I. Shipsey, D. Silvers, J. Sun, A. Svyatkovskiy, F. Wang, W. Xie, L. Xu, N. Parashar, J. Stupak, A. Adair, B. Akgun, Z. Chen, K. M. Ecklund, F. J. M. Geurts, M. Guilbaud, W. Li, B. Michlin, M. Northup, B. P. Padley, R. Redjimi, J. Roberts, J. Rorie, Z. Tu, J. Zabel, B. Betchart, A. Bodek, P. de Barbaro, R. Demina, Y. Eshaq, T. Ferbel, M. Galanti, M. Galanti, A. Garcia-Bellido, J. Han, A. Harel, O. Hindrichs, O. Hindrichs, A. Khukhunaishvili, G. Petrillo, P. Tan, M. Verzetti, S. Arora, A. Barker, J. P. Chou, C. Contreras-Campana, E. Contreras-Campana, D. Duggan, D. Ferencek, Y. Gershtein, R. Gray, E. Halkiadakis, D. Hidas, E. Hughes, S. Kaplan, R. Kunnawalkam Elayavalli, A. Lath, K. Nash, S. Panwalkar, M. Park, S. Salur, S. Schnetzer, D. Sheffield, S. Somalwar, R. Stone, S. Thomas, P. Thomassen, M. Walker, M. Foerster, G. Riley, K. Rose, S. Spanier, A. York, O. Bouhali, A. Castaneda Hernandez, M. Dalchenko, M. De Mattia, A. Delgado, S. Dildick, S. Dildick, R. Eusebi, J. Gilmore, T. Kamon, V. Krutelyov, V. Krutelyov, R. Mueller, I. Osipenkov, Y. Pakhotin, R. Patel, R. Patel, A. Perloff, A. Rose, A. Safonov, A. Tatarinov, K. A. Ulmer, N. Akchurin, C. Cowden, J. Damgov, C. Dragoiu, P. R. Dudero, J. Faulkner, S. Kunori, K. Lamichhane, S. W. Lee, T. Libeiro, S. Undleeb, I. Volobouev, E. Appelt, A. G. Delannoy, S. Greene, A. Gurrola, R. Janjam, W. Johns, C. Maguire, Y. Mao, A. Melo, H. Ni, P. Sheldon, B. Snook, S. Tuo, J. Velkovska, Q. Xu, M. W. Arenton, B. Cox, B. Francis, J. Goodell, R. Hirosky, A. Ledovskoy, H. Li, C. Lin, C. Neu, X. Sun, Y. Wang, E. Wolfe, J. Wood, F. Xia, C. Clarke, R. Harr, P. E. Karchin, C. Kottachchi Kankanamge Don, P. Lamichhane, J. Sturdy, D. A. Belknap, D. Carlsmith, M. Cepeda, S. Dasu, L. Dodd, S. Duric, E. Friis, B. Gomber, M. Grothe, R. Hall-Wilton, M. Herndon, A. Hervé, P. Klabbers, A. Lanaro, A. Levine, K. Long, R. Loveless, A. Mohapatra, I. Ojalvo, T. Perry, G. A. Pierro, G. Polese, T. Ruggles, T. Sarangi, A. Savin, A. Sharma, N. Smith, W. H. Smith, D. Taylor, N. Woods, [Authorinst]The CMS Collaboration

**Affiliations:** Yerevan Physics Institute, Yerevan, Armenia; Institut für Hochenergiephysik der OeAW, Wien, Austria; National Centre for Particle and High Energy Physics, Minsk, Belarus; Universiteit Antwerpen, Antwerpen, Belgium; Vrije Universiteit Brussel, Brussel, Belgium; Université Libre de Bruxelles, Brussel, Belgium; Ghent University, Ghent, Belgium; Université Catholique de Louvain, Louvain-la-Neuve, Belgium; Université de Mons, Mons, Belgium; Centro Brasileiro de Pesquisas Fisicas, Rio de Janeiro, Brazil; Universidade do Estado do Rio de Janeiro, Rio de Janeiro, Brazil; Universidade Estadual Paulista, Universidade Federal do ABC, São Paulo, Brazil; Institute for Nuclear Research and Nuclear Energy, Sofia, Bulgaria; University of Sofia, Sofia, Bulgaria; Institute of High Energy Physics, Beijing, China; State Key Laboratory of Nuclear Physics and Technology, Peking University, Beijing, China; Universidad de Los Andes, Bogota, Colombia; Faculty of Electrical Engineering, Mechanical Engineering and Naval Architecture, University of Split, Split, Croatia; Faculty of Science, University of Split, Split, Croatia; Institute Rudjer Boskovic, Zagreb, Croatia; University of Cyprus, Nicosia, Cyprus; Charles University, Prague, Czech Republic; Academy of Scientific Research and Technology of the Arab Republic of Egypt, Egyptian Network of High Energy Physics, Cairo, Egypt; National Institute of Chemical Physics and Biophysics, Tallinn, Estonia; Department of Physics, University of Helsinki, Helsinki, Finland; Helsinki Institute of Physics, Helsinki, Finland; Lappeenranta University of Technology, Lappeenranta, Finland; DSM/IRFU, CEA/Saclay, Gif-sur-Yvette, France; Laboratoire Leprince-Ringuet, Ecole Polytechnique, IN2P3-CNRS, Palaiseau, France; Institut Pluridisciplinaire Hubert Curien, Université de Strasbourg, Université de Haute Alsace Mulhouse, CNRS/IN2P3, Strasbourg, France; Centre de Calcul de l’Institut National de Physique Nucleaire et de Physique des Particules, CNRS/IN2P3, Villeurbanne, France; Institut de Physique Nucléaire de Lyon, Université de Lyon, Université Claude Bernard Lyon 1, CNRS-IN2P3, Villeurbanne, France; Georgian Technical University, Tbilisi, Georgia; Tbilisi State University, Tbilisi, Georgia; I. Physikalisches Institut, RWTH Aachen University, Aachen, Germany; III. Physikalisches Institut A, RWTH Aachen University, Aachen, Germany; III. Physikalisches Institut B, RWTH Aachen University, Aachen, Germany; Deutsches Elektronen-Synchrotron, Hamburg, Germany; University of Hamburg, Hamburg, Germany; Institut für Experimentelle Kernphysik, Karlsruhe, Germany; Institute of Nuclear and Particle Physics (INPP), NCSR Demokritos, Aghia Paraskevi, Greece; University of Athens, Athens, Greece; University of Ioánnina, Ioannina, Greece; Wigner Research Centre for Physics, Budapest, Hungary; Institute of Nuclear Research ATOMKI, Debrecen, Hungary; University of Debrecen, Debrecen, Hungary; National Institute of Science Education and Research, Bhubaneswar, India; Panjab University, Chandigarh, India; University of Delhi, Delhi, India; Saha Institute of Nuclear Physics, Kolkata, India; Bhabha Atomic Research Centre, Mumbai, India; Tata Institute of Fundamental Research, Mumbai, India; Indian Institute of Science Education and Research (IISER), Pune, India; Institute for Research in Fundamental Sciences (IPM), Tehran, Iran; University College Dublin, Dublin, Ireland; INFN Sezione di Bari, Università di Bari, Politecnico di Bari, Bari, Italy; INFN Sezione di Bologna, Università di Bologna, Bologna, Italy; INFN Sezione di Catania, Università di Catania, CSFNSM, Catania, Italy; INFN Sezione di Firenze, Università di Firenze, Florence, Italy; INFN Laboratori Nazionali di Frascati, Frascati, Italy; INFN Sezione di Genova, Università di Genova, Genoa, Italy; INFN Sezione di Milano-Bicocca, Università di Milano-Bicocca, Milan, Italy; INFN Sezione di Napoli, Università di Napoli ‘Federico II’, Napoli, Italy, Università della Basilicata, Potenza, Italy, Università G. Marconi, Rome, Italy; INFN Sezione di Padova, Università di Padova, Padova, Italy, Università di Trento, Trento, Italy; INFN Sezione di Pavia, Università di Pavia, Pavia, Italy; INFN Sezione di Perugia, Università di Perugia, Perugia, Italy; INFN Sezione di Pisa, Università di Pisa, Scuola Normale Superiore di Pisa, Pisa, Italy; INFN Sezione di Roma, Università di Roma, Rome, Italy; INFN Sezione di Torino, Università di Torino, Turin, Italy, Università del Piemonte Orientale, Novara, Italy; INFN Sezione di Trieste, Università di Trieste, Trieste, Italy; Kangwon National University, Chunchon, Korea; Kyungpook National University, Daegu, Korea; Chonbuk National University, Jeonju, Korea; Chonnam National University, Institute for Universe and Elementary Particles, Kwangju, Korea; Korea University, Seoul, Korea; Seoul National University, Seoul, Korea; University of Seoul, Seoul, Korea; Sungkyunkwan University, Suwon, Korea; Vilnius University, Vilnius, Lithuania; National Centre for Particle Physics, Universiti Malaya, Kuala Lumpur, Malaysia; Centro de Investigacion y de Estudios Avanzados del IPN, Mexico City, Mexico; Universidad Iberoamericana, Mexico City, Mexico; Benemerita Universidad Autonoma de Puebla, Puebla, Mexico; Universidad Autónoma de San Luis Potosí, San Luis Potosí, Mexico; University of Auckland, Auckland, New Zealand; University of Canterbury, Christchurch, New Zealand; National Centre for Physics, Quaid-I-Azam University, Islamabad, Pakistan; National Centre for Nuclear Research, Swierk, Poland; Institute of Experimental Physics, Faculty of Physics, University of Warsaw, Warsaw, Poland; Laboratório de Instrumentação e Física Experimental de Partículas, Lisbon, Portugal; Joint Institute for Nuclear Research, Dubna, Russia; Petersburg Nuclear Physics Institute, Gatchina, Saint Petersburg, Russia; Institute for Nuclear Research, Moscow, Russia; Institute for Theoretical and Experimental Physics, Moscow, Russia; National Research Nuclear University ‘Moscow Engineering Physics Institute’ (MEPhI), Moscow, Russia; P. N. Lebedev Physical Institute, Moscow, Russia; Skobeltsyn Institute of Nuclear Physics, Lomonosov Moscow State University, Moscow, Russia; State Research Center of Russian Federation, Institute for High Energy Physics, Protvino, Russia; Faculty of Physics and Vinca Institute of Nuclear Sciences, University of Belgrade, Belgrade, Serbia; Centro de Investigaciones Energéticas Medioambientales y Tecnológicas (CIEMAT), Madrid, Spain; Universidad Autónoma de Madrid, Madrid, Spain; Universidad de Oviedo, Oviedo, Spain; Instituto de Física de Cantabria (IFCA), CSIC-Universidad de Cantabria, Santander, Spain; CERN, European Organization for Nuclear Research, Geneva, Switzerland; Paul Scherrer Institut, Villigen, Switzerland; Institute for Particle Physics, ETH Zurich, Zurich, Switzerland; Universität Zürich, Zurich, Switzerland; National Central University, Chung-Li, Taiwan; National Taiwan University (NTU), Taipei, Taiwan; Department of Physics, Faculty of Science, Chulalongkorn University, Bangkok, Thailand; Cukurova University, Adana, Turkey; Physics Department, Middle East Technical University, Ankara, Turkey; Bogazici University, Istanbul, Turkey; Istanbul Technical University, Istanbul, Turkey; Institute for Scintillation Materials of National Academy of Science of Ukraine, Kharkov, Ukraine; National Scientific Center, Kharkov Institute of Physics and Technology, Kharkov, Ukraine; University of Bristol, Bristol, UK; Rutherford Appleton Laboratory, Didcot, UK; Imperial College, London, UK; Brunel University, Uxbridge, UK; Baylor University, Waco, USA; The University of Alabama, Tuscaloosa, USA; Boston University, Boston, USA; Brown University, Providence, USA; University of California, Davis, Davis, USA; University of California, Los Angeles, USA; University of California, Riverside, Riverside, USA; University of California, San Diego, La Jolla, USA; University of California, Santa Barbara, Santa Barbara, USA; California Institute of Technology, Pasadena, USA; Carnegie Mellon University, Pittsburgh, USA; University of Colorado Boulder, Boulder, USA; Cornell University, Ithaca, USA; Fermi National Accelerator Laboratory, Batavia, USA; University of Florida, Gainesville, USA; Florida International University, Miami, USA; Florida State University, Tallahassee, USA; Florida Institute of Technology, Melbourne, USA; University of Illinois at Chicago (UIC), Chicago, USA; The University of Iowa, Iowa City, USA; Johns Hopkins University, Baltimore, USA; The University of Kansas, Lawrence, USA; Kansas State University, Manhattan, USA; Lawrence Livermore National Laboratory, Livermore, USA; University of Maryland, College Park, USA; Massachusetts Institute of Technology, Cambridge, USA; University of Minnesota, Minneapolis, USA; University of Mississippi, Oxford, USA; University of Nebraska-Lincoln, Lincoln, USA; State University of New York at Buffalo, Buffalo, USA; Northeastern University, Boston, USA; Northwestern University, Evanston, USA; University of Notre Dame, Notre Dame, USA; The Ohio State University, Columbus, USA; Princeton University, Princeton, USA; University of Puerto Rico, Mayagüez, USA; Purdue University, West Lafayette, USA; Purdue University Calumet, Hammond, USA; Rice University, Houston, USA; University of Rochester, Rochester, USA; Rutgers, The State University of New Jersey, Piscataway, USA; University of Tennessee, Knoxville, USA; Texas A&M University, College Station, USA; Texas Tech University, Lubbock, USA; Vanderbilt University, Nashville, USA; University of Virginia, Charlottesville, USA; Wayne State University, Detroit, USA; University of Wisconsin, Madison, USA; CERN, Geneva, Switzerland

## Abstract

The cross section for $\mathrm{t}\overline{{\mathrm{t}}}$ production in the all-jets final state is measured in pp collisions at a centre-of-mass energy of 8 $\,\text {TeV}$ at the LHC with the CMS detector, in data corresponding to an integrated luminosity of 18.4 $\,\text {fb}^{-1}$. The inclusive cross section is found to be $275.6 \pm 6.1 \,\text {(stat)} \pm 37.8 \,\text {(syst)} \pm 7.2 \,\text {(lumi)}$ $\text {\,pb}$. The normalized differential cross sections are measured as a function of the top quark transverse momenta, $p_{\mathrm {T}}$, and compared to predictions from quantum chromodynamics. The results are reported at detector, parton, and particle levels. In all cases, the measured top quark $p_{\mathrm {T}}$ spectra are significantly softer than theoretical predictions.

## Introduction

The top quark is an important component of the standard model (SM), especially because of its large mass, and its properties are critical for the overall understanding of the theory. Measurements of the top quark–antiquark pair (${\mathrm{t}}\overline{{\mathrm{t}}}$) production cross section test the predictions of quantum chromodynamics (QCD), constrain QCD parameters, and are sensitive to physics beyond the SM. The ${\mathrm{t}}\overline{{\mathrm{t}}}$ process is also the dominant SM background to many searches for new physical phenomena, and its precise measurement is essential for claiming new discoveries.

The copious top quark data samples produced at the CERN LHC enable measurements of the ${\mathrm{t}}\overline{{\mathrm{t}}}$ production rate in extended parts of the phase space, and differentially as a function of the kinematic properties of the ${\mathrm{t}}\overline{{\mathrm{t}}}$ system. Inclusive and differential cross section measurements from proton-proton (pp) collisions at centre-of-mass energies of 7 and 8 $\,\text {TeV}$ have been reported by the ATLAS [1–11] and CMS collaborations [12–24]. These are significantly more precise than the measurements of ${\mathrm{t}}\overline{{\mathrm{t}}}$ production in proton-antiproton collisions performed at the Tevatron [25]. In this paper, we report new results from pp collision data at $\sqrt{s}=8~\,\text {TeV}$, collected with the CMS detector. Measurements of the ${\mathrm{t}}\overline{{\mathrm{t}}}$ inclusive cross section and the normalized differential cross sections are presented for the first time in the all-jets final state at this collision energy. The results are compared to QCD predictions, and are in agreement with other measurements in different decay channels.

Top quarks decay almost exclusively into a W boson and a b quark. Events in which both W bosons from the ${\mathrm{t}}\overline{{\mathrm{t}}}$ decay produce a pair of light quarks constitute the so-called all-jets channel. As a result, the final state consists of at least six partons (more are possible from initial- and final-state radiation), two of which are b quarks. Despite the large number of combinatorial possibilities, it is possible to fully reconstruct the kinematical properties of the ${\mathrm{t}}\overline{{\mathrm{t}}}$ decay products, unlike in the leptonic channels where the presence of one or two neutrinos makes the full event interpretation ambiguous. However, the presence of a large background from multijet production, and the larger number of jets in the final state make the measurement of the ${\mathrm{t}}\overline{{\mathrm{t}}}$ cross section in the all-jets final state more uncertain compared to the leptonic channels. Nevertheless, a high-purity signal sample can be selected, which increases significantly the signal-over-background ratio compared to previous measurements in this decay channel [21, 26, 27].

## The CMS detector

The central feature of the CMS apparatus is a superconducting solenoid of 6 $\text {\,m}$ internal diameter, providing a magnetic field of 3.8 $\text {\,T}$. Within the solenoid volume are a silicon pixel and strip tracker, a lead tungstate crystal electromagnetic calorimeter, and a brass and scintillator hadron calorimeter. Extensive forward calorimetry (pseudorapidity $|\eta |>3.0$) complements the coverage provided by the barrel ($|\eta |<1.3$) and endcap ($1.3<|\eta |<3.0$) detectors. Muons are measured in gas-ionization detectors embedded in the steel flux-return yoke outside the solenoid. The first level of the CMS trigger system, composed of custom hardware processors, uses information from the calorimeters and muon detectors to select the most interesting events in a fixed time interval of less than 4 $\upmu$s. The high-level trigger (HLT) processor farm further decreases the event rate from around 100 $\text {\,kHz}$ to around 300 $\text {\,Hz}$, before data storage. A detailed description of the CMS apparatus, together with the definition of the coordinate system used and the relevant kinematic variables, can be found in Ref. [28].

## Event simulation

The ${\mathrm{t}}\overline{{\mathrm{t}}}$ events are simulated using the leading-order (LO) MadGraph (v. 5.1.5.11) event generator [29], which incorporates spin correlations through the madspin [30] package and the simulation of up to three additional partons. The value of the top quark mass is set to $m_{{\mathrm{t}}}=172.5~\,\text {GeV}$ and the proton structure is described by the parton distribution functions (PDFs) from CTEQ6L1 [31]. The generated events are subsequently processed with pythia (v. 6.426) [32] which utilizes tune Z2* for parton showering and hadronization, and the MLM prescription [33] is used for matching of matrix element jets to those from parton shower. The pythia Z2* tune is derived from the Z1* tune [34], which uses the CTEQ5L PDF [31], whereas Z2* adopts CTEQ6L [31]. The CMS detector response is simulated using Geant4 (v. 9.4) [35].

In addition to the MadGraph simulation, predictions obtained with the next-to-leading-order (NLO) generators mc@nlo (v. 3.41) [36] and powheg (v. 1.0 r1380) [37] are also compared to the measurements. While powheg and mc@nlo are formally equivalent up to NLO accuracy, they differ in the techniques used to avoid double counting of the radiative corrections when interfacing with the parton shower generators. Two different powheg samples are used: one uses pythia and the other herwig (v. 6.520) [38] for parton showering and hadronization. The events generated with mc@nlo are interfaced with herwig. The herwig AUET2 tune [39] is used to model the underlying event in the powheg$+$herwig sample, while the default tune is used in the mc@nlo$+$herwig sample. The proton structure is described by the PDF sets CT10 [40] and CTEQ6M [31] for powheg and mc@nlo, respectively. The QCD multijet events are simulated using MadGraph (v. 5.1.3.2) interfaced with pythia (v. 6.424).

## Event reconstruction and selection

### Jet reconstruction

Jets are reconstructed with the anti-$k_{\mathrm {T}}$ clustering algorithm [41, 42] with a distance parameter of 0.5. The input to the jet clustering algorithm is the collection of particle candidates that are reconstructed with the particle-flow (PF) algorithm [43, 44]. In the PF event reconstruction all stable particles in the event, i.e. electrons, muons, photons, and charged and neutral hadrons, are reconstructed as PF candidates using a combination of all of the subdetector information to obtain an optimal determination of their directions, energies, and types. All the reconstructed vertices in the event are ordered according to the sum of squared transverse momenta ($p_{\mathrm {T}}$) of tracks used to reconstruct it and the vertex with the largest sum is considered the primary one, while all the rest are considered as pileup vertices. In order to mitigate the effect of multiple interactions in the same bunch crossing (pileup), charged PF candidates that are unambiguously associated with pileup vertices are removed prior to the jet clustering. This procedure is called charged-hadron subtraction (CHS) [45]. An offset correction is applied for the additional energy inside of the jet due to neutral hadrons or photons from pileup. The resulting jets require a small residual energy correction, mostly due to the thresholds for reconstructed tracks and clusters in the PF algorithm and reconstruction inefficiencies [45].

The identification of jets that likely originate from the hadronization of b quarks is done with the “combined secondary vertex” (CSV) b tagger [46]. The CSV algorithm combines the information from track impact parameters and identified secondary vertices within a given jet, and provides a continuous discriminator output.

### Trigger

The data used for this measurement were collected with a multijet trigger event selection (path) which, from the HLT, required at least four jets reconstructed from calorimetric information with a $p_{\mathrm {T}}$ threshold of $50~\,\text {GeV}$ and $|\eta |<3.0$. The hardware trigger required the presence of two central ($|\eta |<3.0$) jets above various $p_{\mathrm {T}}$ thresholds (52–64 $\,\text {GeV}$), or the presence of four central jets with lower $p_{\mathrm {T}}$ thresholds (32–40 $\,\text {GeV}$), or the scalar sum of all jets $p_{\mathrm {T}}$ to be greater than 125 or 175 $\,\text {GeV}$. The various thresholds were adjusted within the quoted ranges according to the instantaneous luminosity. The trigger paths employed were unprescaled for a larger part of the run, yielding a data sample corresponding to an integrated luminosity of 18.4 $\,\text {fb}^{-1}$.

**Fig. 1:**
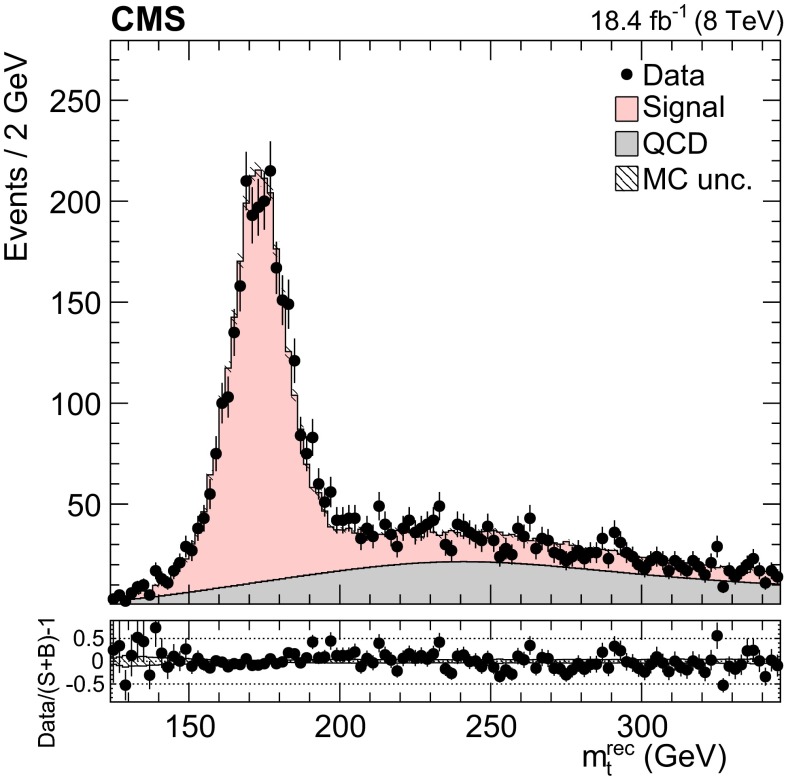
Distribution of the reconstructed top quark mass after the kinematic fit. The normalizations of the ${\mathrm{t}}\overline{{\mathrm{t}}}$ signal and the QCD multijet background are taken from the template fit to the data. The *bottom panel* shows the fractional difference between the data and the sum of signal and background predictions, with the *shaded band* representing the MC statistical uncertainty

**Fig. 2:**
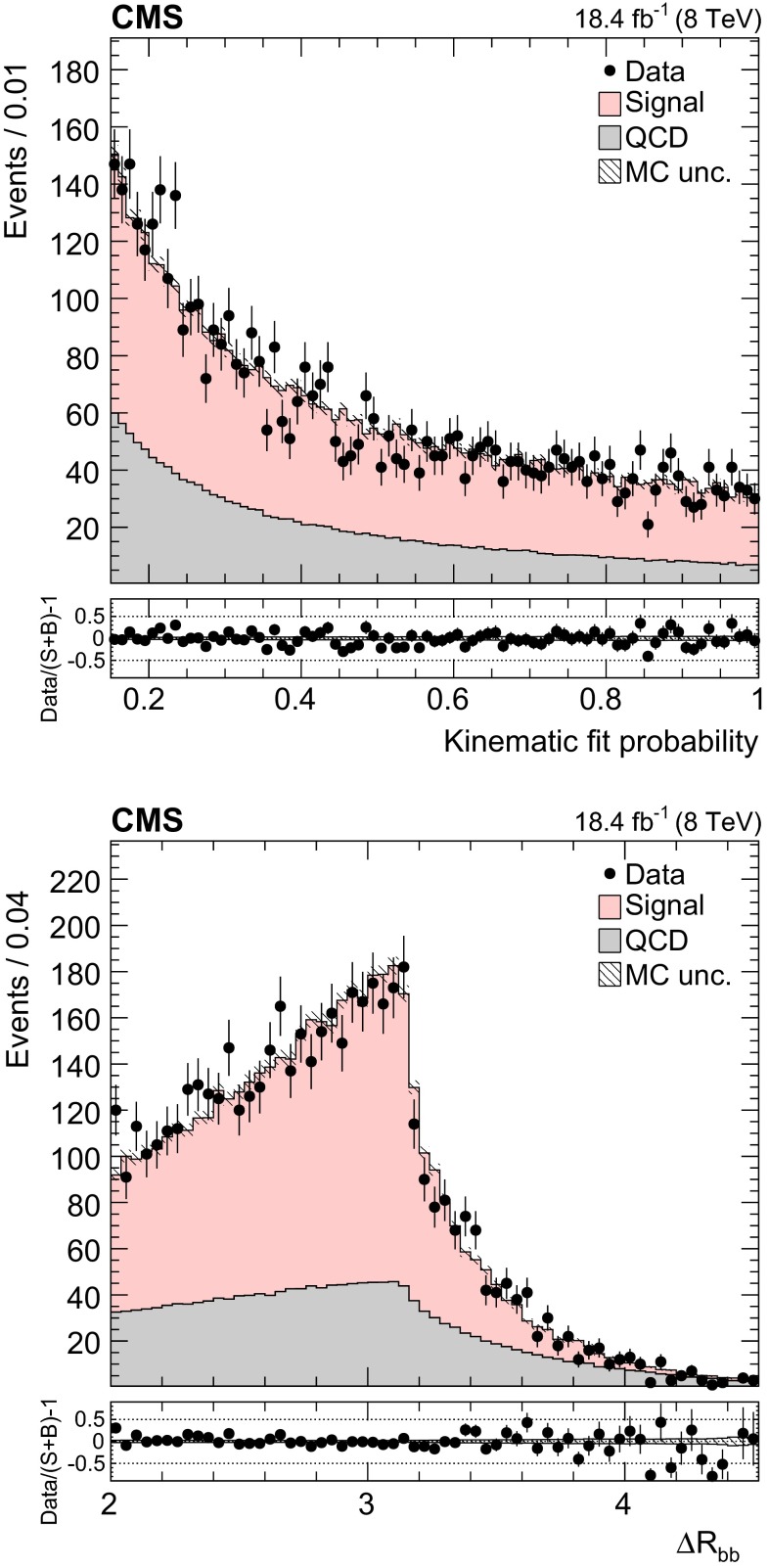
Distribution of the kinematic fit probability (*top*). Distribution of the distance between the reconstructed b partons in the $\eta$–$\phi$ plane (*bottom*). The normalizations of the ${\mathrm{t}}\overline{{\mathrm{t}}}$ signal and the QCD multijet background are taken from the template fit to the data. The *bottom panels* show the fractional difference between the data and the sum of signal and background predictions, with the *shaded band* representing the MC statistical uncertainty

### Selection and kinematic top quark pair reconstruction

Selected events are required to contain at least six reconstructed jets with $p_{\mathrm {T}} >40~\,\text {GeV}$ and $|\eta |<2.4$ (jets are required to be within the tracker acceptance in order to apply the CHS), with at least four of the jets having $p_{\mathrm {T}} >60~\,\text {GeV}$ (so that the trigger efficiency is greater than 80 % and the data-to-simulation correction factor smaller than 10 %). Among the six jets with the highest $p_{\mathrm {T}}$ (leading jets), at least two must be identified as coming from b hadronization by the CSV algorithm at the medium working point (CSVM), with a typical b quark identification efficiency of 70 % and misidentification probability for light quarks of 1.4 %, and these are considered the most probable b jet candidates. If there are more than two such jets, which happens in approximately 2 % of the events, then the two with the highest $p_{\mathrm {T}}$ are chosen. To select events compatible with the ${\mathrm{t}}\overline{{\mathrm{t}}}$ hypothesis, and to improve the resolution of the reconstructed quantities, a kinematic fit is performed that utilizes the constraints of the ${\mathrm{t}}\overline{{\mathrm{t}}}$ decay. A $\chi ^2$ fit is performed, starting with the reconstructed jet four-momenta, which are varied within their experimental $p_{\mathrm {T}}$ and angular resolutions, imposing a W boson mass constraint (80.4 $\,\text {GeV}$  [47]) on the light-quark pairs, and requiring that the top quark and antiquark have equal mass. Out of all the possible combinations from the six input jets, the algorithm returns the one with the smallest $\chi ^2$ and the resulting parton four momenta, which are used to compute the reconstructed top quark mass ($m_{{\mathrm{t}}}^\text {rec}$). The probability of the converged kinematic fit is required to be greater than 0.15. Overall, the kinematic fit requirements select approximately 5 % (2 %) of the ${\mathrm{t}}\overline{{\mathrm{t}}}$ (background) events. The distance in the $\eta$–$\phi$ space between the two b quark candidates must be $\Delta R_{{\mathrm{b}}{\mathrm{b}}}=\sqrt{{(\Delta \eta _{{\mathrm{b}}{\mathrm{b}}})^2+(\Delta \phi _{{\mathrm{b}}{\mathrm{b}}})^2}}>2.0$, which has an efficiency of roughly 75 % (50 %) on ${\mathrm{t}}\overline{{\mathrm{t}}}$ (background) events. The last two requirements are applied to select events with unambiguous top quark pair interpretation and to suppress the QCD background that originates from gluon splitting into collinear b quarks [48].

**Fig. 3:**
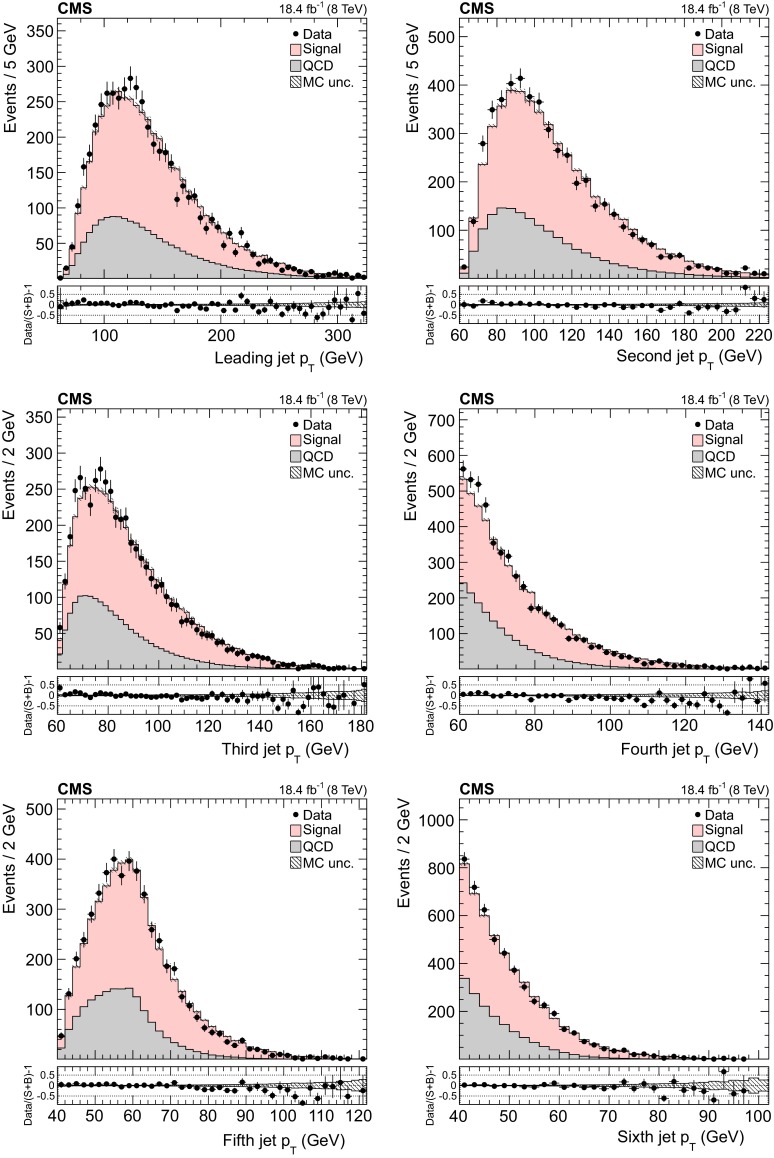
Distribution of the $p_{\mathrm {T}}$ of the six leading jets. The normalization of the ${\mathrm{t}}\overline{{\mathrm{t}}}$ signal and the QCD multijet background are taken from the template fit to the data. The *bottom panels* show the fractional difference between the data and the sum of signal and background predictions, with the *shaded band* representing the MC statistical uncertainty

**Fig. 4:**
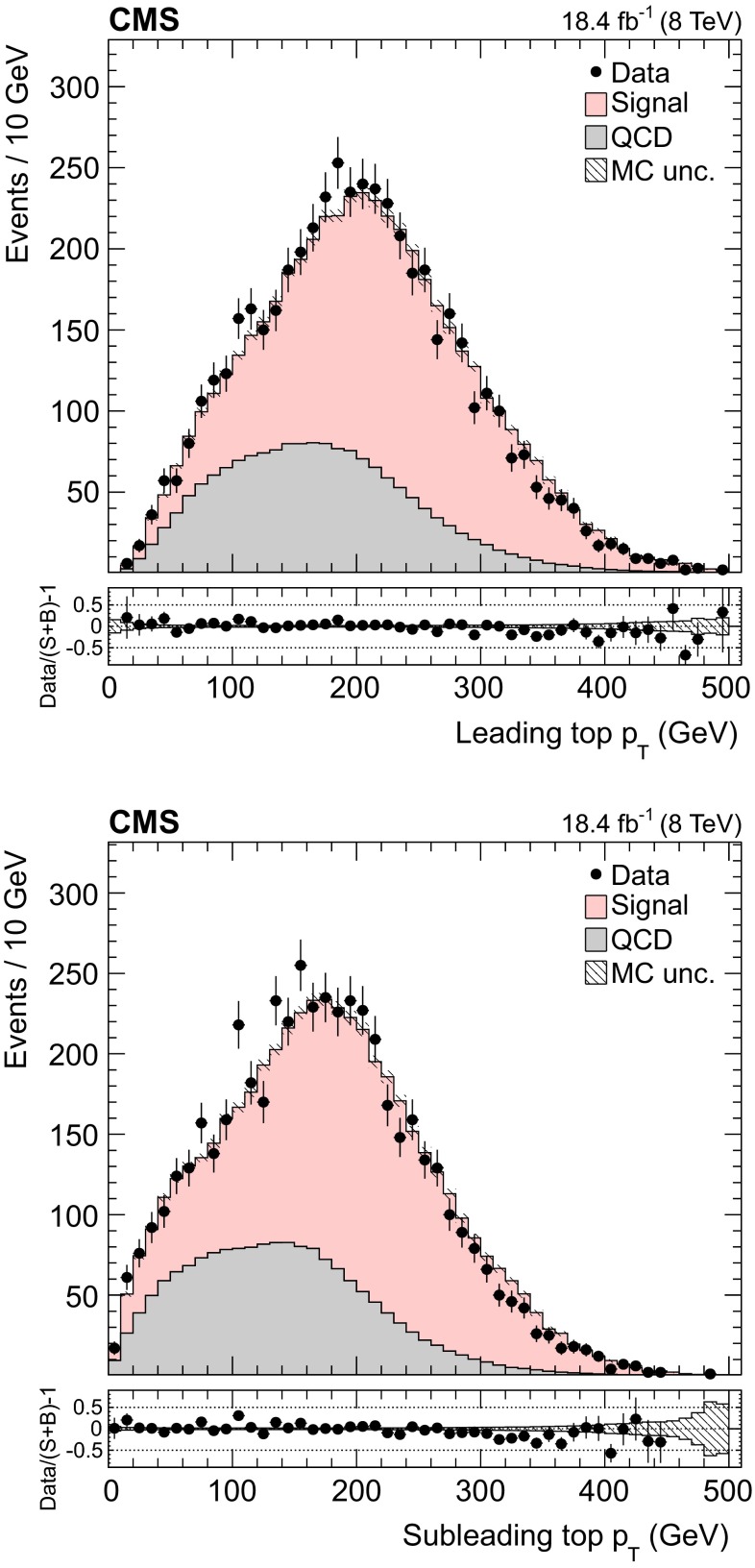
Distribution of the leading (*top*) and subleading (*bottom*) reconstructed top quark $p_{\mathrm {T}}$. The normalizations of the ${\mathrm{t}}\overline{{\mathrm{t}}}$ signal and the QCD multijet background are taken from the template fit to the data. The *bottom panels* show the fractional difference between the data and the sum of signal and background predictions, with the *shaded band* representing the MC statistical uncertainty

**Fig. 5:**
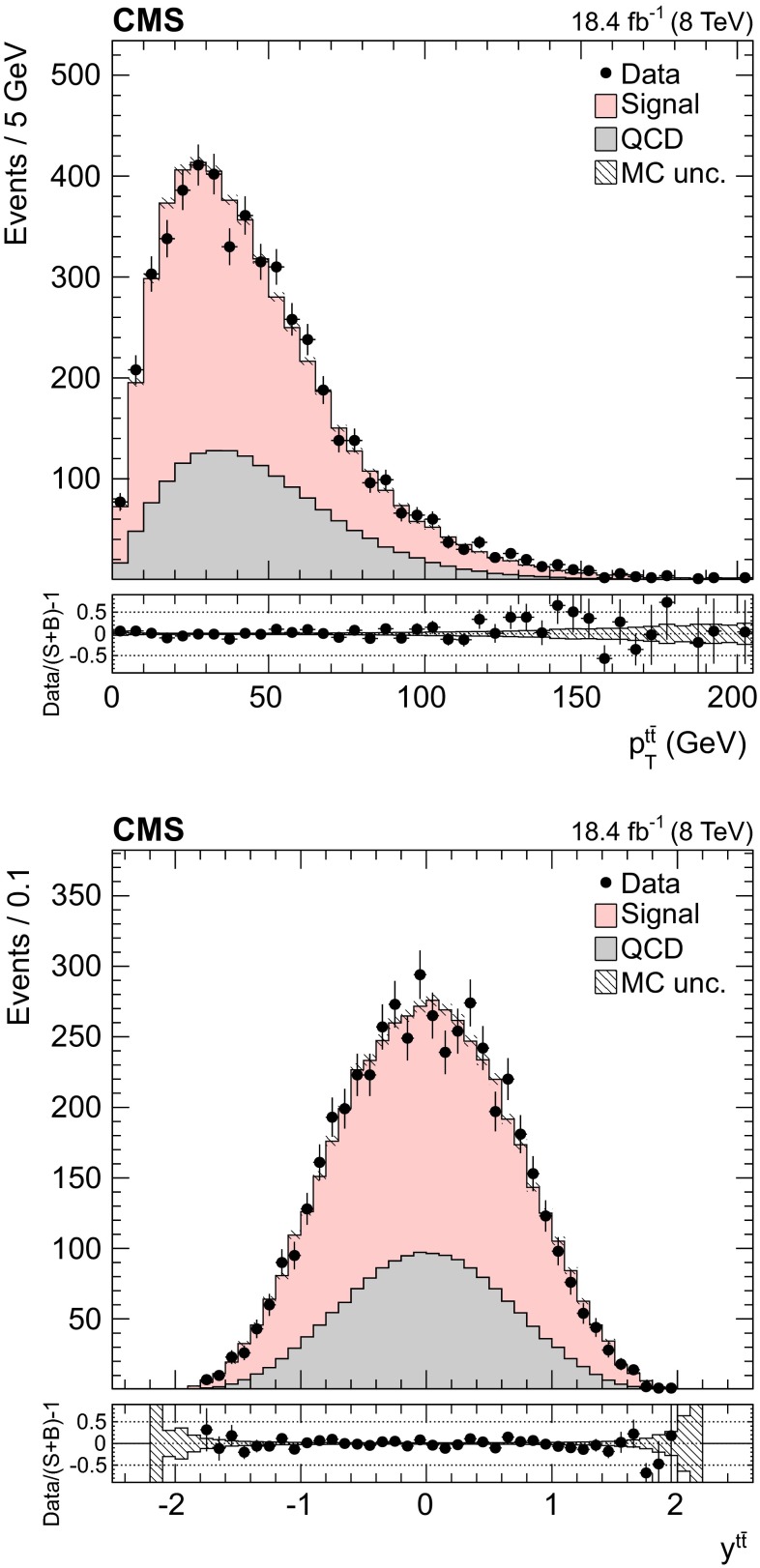
Distribution of the $p_{\mathrm {T}}$ (*top*) and the rapidity (*bottom*) of the reconstructed top quark pair. The normalizations of the ${\mathrm{t}}\overline{{\mathrm{t}}}$ signal and the QCD multijet background are taken from the template fit to the data. The *bottom panels* show the fractional difference between the data and the sum of signal and background predictions, with the *shaded band* representing the MC statistical uncertainty

## Signal extraction

The background to the ${\mathrm{t}}\overline{{\mathrm{t}}}$ signal is dominated by the QCD multijet production process, while the other backgrounds, such as the associated production of vector bosons with jets, are negligible. Due to the limited size of the Monte Carlo (MC) simulated samples, the background is determined directly from the data. A QCD-dominated event sample is selected with the trigger and offline requirements described in Sect. 4.3 and requiring zero CSVM b tagged jets. In these events the most probable b quark candidates are determined by the kinematic fit. The resulting sample contains a negligible fraction of ${\mathrm{t}}\overline{{\mathrm{t}}}$ events ($<$1 %) and is treated exactly like the signal sample. After applying the $\Delta R_{{\mathrm{b}}{\mathrm{b}}}>2.0$ and the fit probability requirements, the reconstructed top-like kinematic properties of events with no b jet are very similar to those with two b jets (confirmed using simulated QCD events). We use this QCD-dominated control sample to extract the shape (templates) of the various kinematic observables. The number of ${\mathrm{t}}\overline{{\mathrm{t}}}$ events (signal yield) is extracted from a template fit of $m_{{\mathrm{t}}}^\text {rec}$ to the data using parametrized shapes for signal and background distributions, where the signal shape is taken from the ${\mathrm{t}}\overline{{\mathrm{t}}}$ simulation and the QCD shape is taken from the control data sample described above. The background and signal yields are determined via a maximum likelihood fit to the $m_{{\mathrm{t}}}^\text {rec}$ distribution and are used to normalize the corresponding samples. Figures 1 and 2 show the fitted mass and the kinematic fit probability and $\Delta R_{{\mathrm{b}}{\mathrm{b}}}$ distributions. The $p_{\mathrm {T}}$ distributions of the six leading jets is shown in Fig. 3. From the output of the kinematic fit one can reconstruct the two top quark candidates, whose $p_{\mathrm {T}}$ are shown in Fig. 4, and the properties of the ${\mathrm{t}}\overline{{\mathrm{t}}}$ system ($p_{\mathrm {T}}$, rapidity *y*) are shown in Fig. 5. Overall, the data sample is dominated by signal events, and the data are in agreement with the fit results. The jet $p_{\mathrm {T}}$ spectra in data appear to be systematically softer than in the simulation, in agreement with the observations in Ref. [24], related to a softer measured top quark $p_{\mathrm {T}}$ spectrum.

**Fig. 6:**
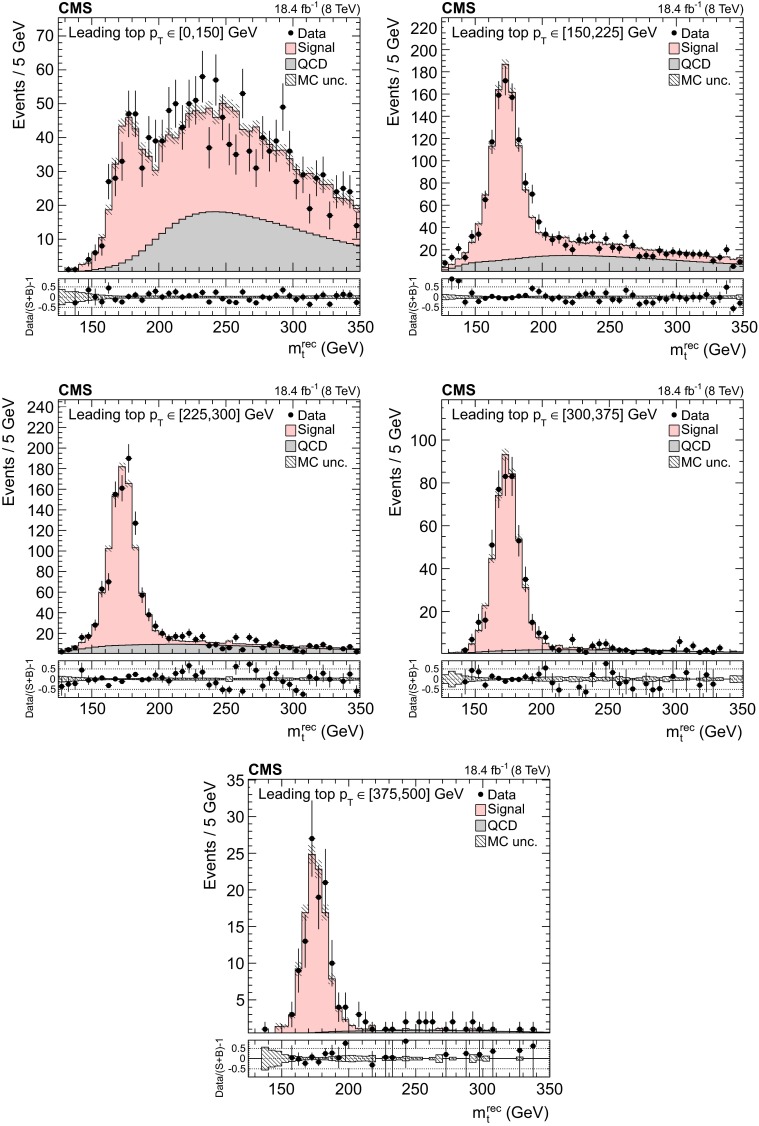
Distribution of the reconstructed top quark mass after the kinematic fit in bins of the leading reconstructed top quark $p_{\mathrm {T}}$. The normalizations of the ${\mathrm{t}}\overline{{\mathrm{t}}}$ signal and the QCD multijet background are taken from the template fit to the data. The *bottom panels* show the fractional difference between the data and the sum of signal and background predictions, with the *shaded band* representing the MC statistical uncertainty

## Systematic uncertainties

The measurement of the ${\mathrm{t}}\overline{{\mathrm{t}}}$ cross section is affected by several sources of systematic uncertainty, both experimental and theoretical, which are described below and summarized in Table 1. The quoted values refer to the inclusive measurement, with small variations observed in the bins of the differential measurement presented in Sect. 7.2.

**Table 1:** Fractional uncertainties in the inclusive ${\mathrm{t}}\overline{{\mathrm{t}}}$ production cross section

Source	%
Background modeling	$\pm 4.9$
JES	$-7.0,\,+6.8$
JER	$\pm 3.5$
b tagging	$\pm 7.3$
Trigger efficiency	$-2.2,\,+2.0$
Underlying event	$\pm 4.4$
Matching partons to showers	$-4.2,\,+2.4$
Factorization and renormalization scales	$-0.5,\,+3.8$
Color reconnection	$\pm 1.4$
Parton distribution function	$\pm 1.5$
Hadronization	$\pm 2.0$
Total systematic uncertainty	${\pm }13.7$
Statistical uncertainty	$\pm 2.3$
Integrated luminosity	$\pm 2.6$

**Fig. 7:**
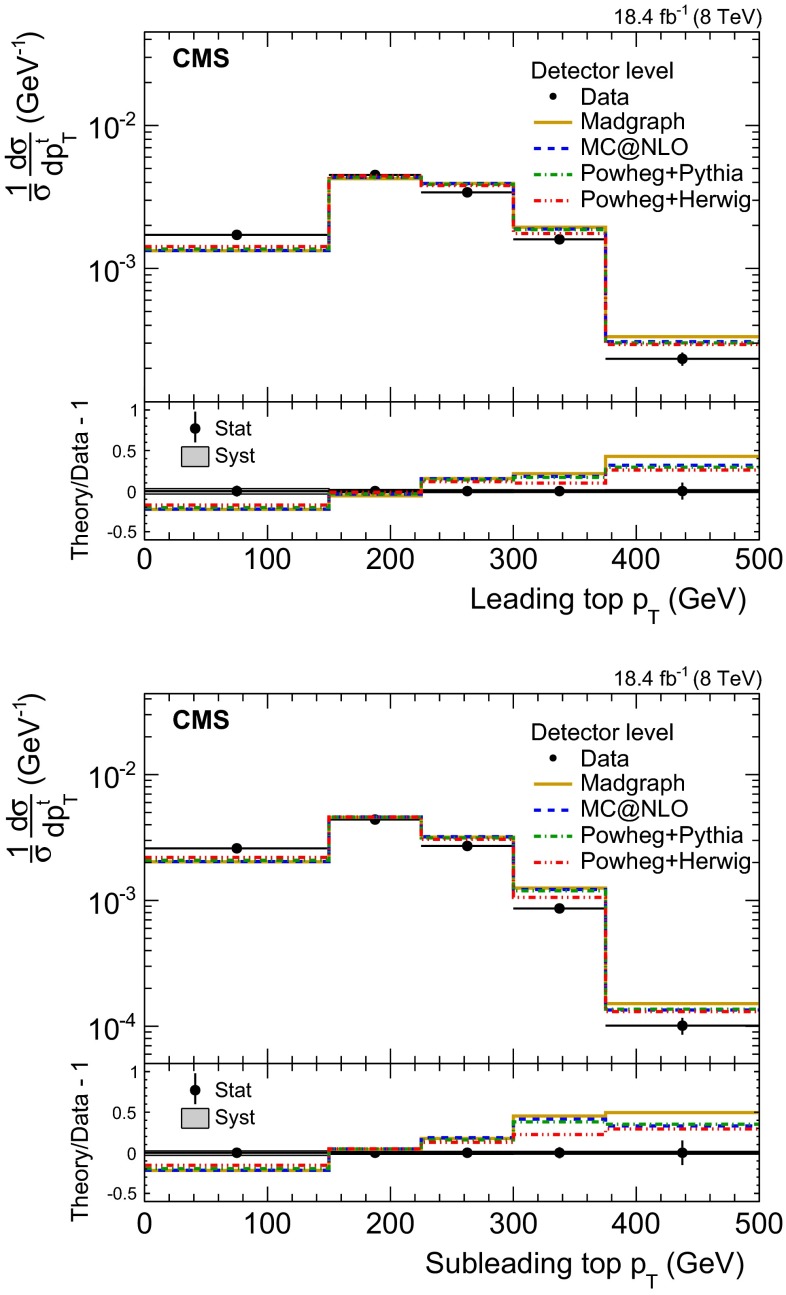
Normalized fiducial differential cross section of the ${\mathrm{t}}\overline{{\mathrm{t}}}$ production as a function of the leading (*top*) and subleading (*bottom*) reconstructed top quark $p_{\mathrm {T}}$ (detector level). The *bottom panels* show the fractional difference between various MC predictions and the data. Statistical uncertainties are shown with *error bars*, and systematic uncertainties with the *shaded band*

**Table 2:** Normalized differential ${\mathrm{t}}\overline{{\mathrm{t}}}$ cross section as a function of the $p_{\mathrm {T}}$ of the leading ($p_{\mathrm {T}} ^{(1)}$) and subleading ($p_{\mathrm {T}} ^{(2)}$) top quarks or antiquarks. The results are presented at detector level in the visible phase space

$p_{\mathrm {T}}$ bin range ($\text {GeV}$ )	$\frac{1}{\sigma } {\mathrm{d}}\sigma /{\mathrm{d}}p_{\mathrm {T}} ^{(1)} (\text {GeV} ^{-1})$	Stat (%)	Syst (%)
[0, 150]	$1.72\times 10^{-3}$	$\pm 6.7$	$\pm 3.7$
[150, 225]	$4.51\times 10^{-3}$	$\pm 3.7$	$\pm 2.0$
[225, 300]	$3.41\times 10^{-3}$	$\pm 3.9$	$\pm 1.8$
[300, 375]	$1.60\times 10^{-3}$	$\pm 5.3$	$\pm 1.6$
[375, 500]	$2.33\times 10^{-4}$	$\pm 10.4$	$\pm 1.7$

$p_{\mathrm {T}}$ bin range ($\text {GeV}$ )	$\frac{1}{\sigma } {\mathrm{d}}\sigma /{\mathrm{d}}p_{\mathrm {T}} ^{(2)} (\text {GeV} ^{-1})$	Stat (%)	Syst (%)
[0, 150]	$2.59\times 10^{-3}$	$\pm 3.9$	$\pm 3.3$
[150, 225]	$4.39\times 10^{-3}$	$\pm 3.4$	$\pm 1.9$
[225, 300]	$2.71\times 10^{-3}$	$\pm 4.1$	$\pm 1.9$
[300, 375]	$8.64\times 10^{-4}$	$\pm 7.0$	$\pm 1.8$
[375, 500]	$1.01\times 10^{-4}$	$\pm 15.2$	$\pm 1.7$

**Fig. 8:**
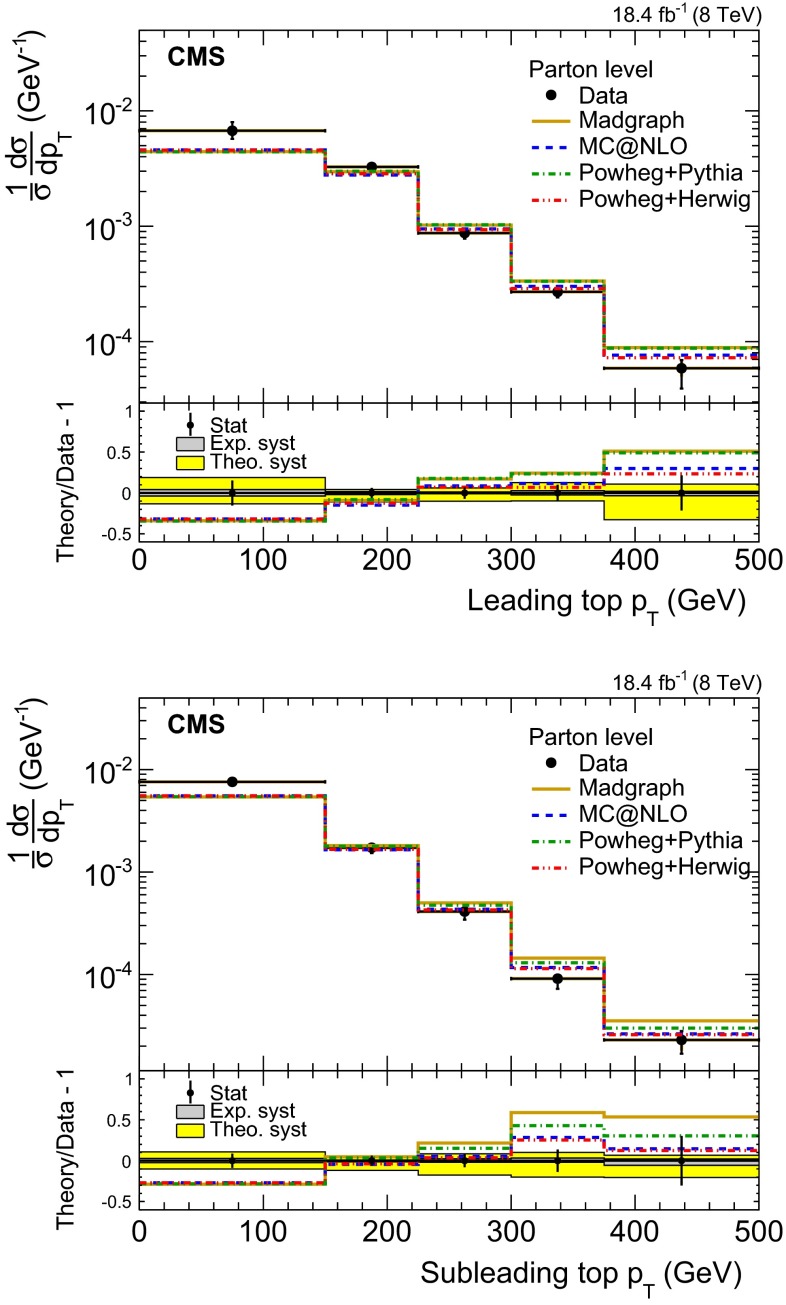
Normalized differential cross section of the ${\mathrm{t}}\overline{{\mathrm{t}}}$ production at parton level as a function of the leading (*top*) and subleading (*bottom*) top quark $p_{\mathrm {T}}$. The *bottom panels* show the fractional difference between various MC predictions and the data. Statistical uncertainties are shown with *error bars*, while theoretical (*theo.*) and experimental (*exp.*) systematic uncertainties with the *shaded bands*

- **Background modeling:** the QCD $m_{{\mathrm{t}}}^\text {rec}$ template shape derived from the data control sample is varied according to the uncertainty of the method evaluated with simulated events, which impacts the extracted signal yield moderately (4.9 %).
- **Trigger efficiency:** the efficiency of the trigger path is taken from the simulation and corrected with an event-by-event scale factor ($\mathrm {SF}_\text {trig}$), calculated from data independent samples, that depends on the fourth jet $p_{\mathrm {T}}$. In the phase space of the measurement, the $\mathrm {SF}_\text {trig}$ is greater than 0.83 and on average 0.96. The associated uncertainty is conservatively defined as $(1-\mathrm {SF}_\text {trig})/2$ and has a small impact (2.0 %) on the cross section.
- **Jet energy scale and resolution:** the jet energy scale (JES) and jet resolution (JER) uncertainties have significant impacts on the measured cross section due to the relatively high $p_{\mathrm {T}}$ requirements on the fourth and sixth of the leading jets. In the simulated events, jets are shifted (smeared) according to the $p_{\mathrm {T}}$- and $\eta$-dependent JES (JER) uncertainty, prior to the kinematic fit, and the full event interpretation is repeated. The JES (JER) has a dominant (small) effect on the cross section measurement of 7.0 % (3.5 %). In addition, the JES/JER uncertainties affect the signal template, with a negligible impact ($\approx$1 %) on the cross section measurement.
- ** b tagging:** the performance of the b tagger has a dominant effect on the signal acceptance because the selected events are required to have at least two jets satisfying the CSVM requirement. An event-by-event scale factor ($\mathrm {SF}_\text {btag}$) is applied to the simulation, which accounts for the discrepancies between data and simulation in the efficiency of tagging true b jets and in the misidentification rate [46]. The average value of $\mathrm {SF}_\text {btag}$ is 0.99. The uncertainty in the $\mathrm {SF}_\text {btag}$ is taken into account by weighting each event with the shifted value of $\mathrm {SF}_\text {btag}$ which results in a cross section uncertainty of 7.3 %. This is the leading systematic uncertainty.
- **Integrated luminosity:** the uncertainty on the integrated luminosity is estimated to be 2.6 % [49].
- **Matching partons to showers:** the impact of the choice of the scale that separates the description of jet production via matrix elements or parton shower in MadGraph is studied by changing its reference value of 20 to 40 and 10 $\,\text {GeV}$, resulting in an asymmetric effect of $-4.2,\,+2.4$ % on the cross section.
- **Renormalization and factorization scales: ** the uncertainty in modelling of the hard-production process is assessed through changes in the renormalization and factorization scales in the MadGraph sample by factors of two and half, relative to their common nominal value, which is set to the *Q* of the hard process. In MadGraph, *Q* is defined by $Q^2 = m^2_{{\mathrm{t}}} + \Sigma p^2_{\mathrm {T}}$, where the sum is over all additional final state partons in the matrix element calculations. The effect on the measured cross section is moderate and asymmetric ($-0.5,\,+3.8~\%$).
- **Parton distribution functions: ** following the PDF4LHC prescription [50, 51], the uncertainty on the cross section is estimated to be 1.5 %, taking the largest deviation on the signal acceptance from all the considered PDF eigenvectors.

**Table 3:** Normalized differential ${\mathrm{t}}\overline{{\mathrm{t}}}$ cross section as a function of the $p_{\mathrm {T}}$ of the leading ($p_{\mathrm {T}} ^{(1)}$) and subleading ($p_{\mathrm {T}} ^{(2)}$) top quarks or antiquarks. The results are presented at parton level in the full phase space

$p_{\mathrm {T}}$ bin range ($\text {GeV}$ )	$\frac{1}{\sigma } {\mathrm{d}}\sigma /{\mathrm{d}}p_{\mathrm {T}} ^{(1)} (\text {GeV} ^{-1})$	Stat (%)	Exp. syst (%)	Theo. syst (%)
[0, 150]	$6.72\times 10^{-3}$	$\pm 10.8$	$-3.7, +4.1$	$-9.7, +14.8$
[150, 225]	$3.27\times 10^{-3}$	$\pm 4.3$	$-2.0, +1.8$	$-9.0, +2.5$
[225, 300]	$8.73\times 10^{-4}$	$\pm 5.0$	$-0.8, +1.2$	$-9.3, +4.9$
[300, 375]	$2.70\times 10^{-4}$	$\pm 7.1$	$-2.3, +2.7$	$-7.5, +9.9$
[375, 500]	$5.88\times 10^{-5}$	$\pm 15.2$	$-3.3, +1.9$	$-29.4, +9.0$

$p_{\mathrm {T}}$ bin range ($\text {GeV}$ )	$\frac{1}{\sigma } {\mathrm{d}}\sigma /{\mathrm{d}}p_{\mathrm {T}} ^{(2)} (\text {GeV} ^{-1})$	Stat (%)	Exp. syst (%)	Theo. syst (%)
[0, 150]	$7.59\times 10^{-3}$	$\pm 6.2$	$-2.5, +2.7$	$-7.6, +8.1$
[150, 225]	$1.73\times 10^{-3}$	$\pm 4.4$	$-1.3, +0.7$	$-10.5, +4.7$
[225, 300]	$4.12\times 10^{-4}$	$\pm 5.6$	$-1.8, +2.2$	$-15.7, +6.2$
[300, 375]	$9.11\times 10^{-5}$	$\pm 9.7$	$-1.9, +3.3$	$-18.1, +7.0$
[375, 500]	$2.30\times 10^{-5}$	$\pm 21.4$	$-5.6, +2.0$	$-15.0, +4.7$
- **Non-perturbative QCD: ** the impact of non-perturbative QCD effects is estimated by studying various tunes of the pythia shower model that predict different underlying event (UE) activity and strength of the color reconnection (CR), namely, the Perugia 2011, Perugia 2011 mpiHi, and Perugia 2011 Tevatron tunes, described in Ref. [52], were used. The effect on the measured cross section is moderate: 4.4 % for the UE and 1.4 % for the CR.
- **Hadronization model: ** the effect of the hadronization model on the signal efficiency is estimated by comparing the predictions from the mc@nlo$+$herwig and powheg$+$pythia simulations, and it amounts to 2 %.

**Fig. 9:**
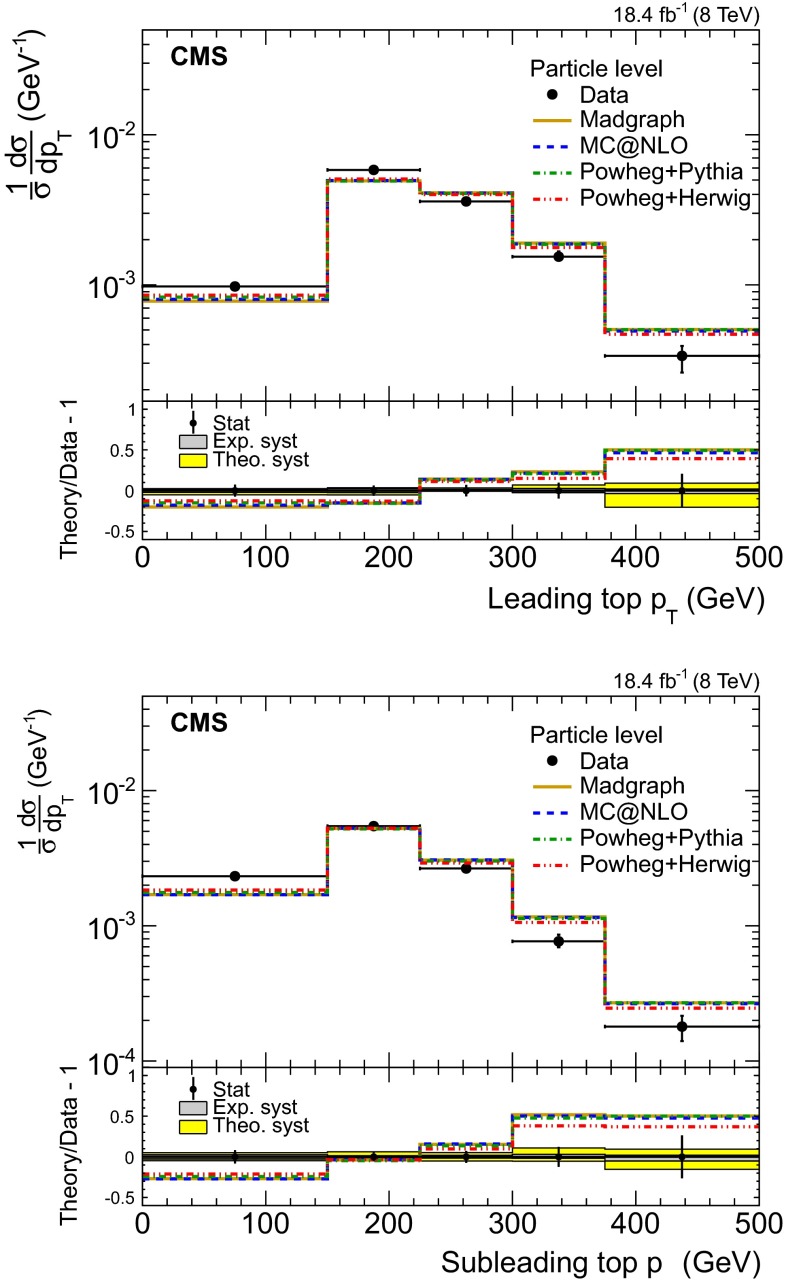
Normalized differential cross section of the ${\mathrm{t}}\overline{{\mathrm{t}}}$ production at particle level as a function of the leading (*top*) and subleading (*bottom*) top quark $p_{\mathrm {T}}$. The *bottom panels* show the fractional difference between various MC predictions and the data. Statistical uncertainties are shown with *error bars*, while theoretical (*theo*.) and experimental (*exp*.) systematic uncertainties with the *shaded bands*

## Results

### Inclusive cross section

The signal yield ($N_{{\mathrm{t}}\overline{{\mathrm{t}}}}$), extracted as described in Sect. 5, is used to compute the inclusive ${\mathrm{t}}\overline{{\mathrm{t}}}$ production cross section, according to the formula

1 $$\begin{aligned} \sigma _{{\mathrm{t}}\overline{{\mathrm{t}}}}=\frac{N_{{\mathrm{t}}\overline{{\mathrm{t}}}}}{(\mathcal {A}\epsilon )\,\mathcal {L}}, \end{aligned}$$

where $(\mathcal {A}\,\epsilon )$ is the simulated signal acceptance times efficiency in the measurement phase space (${\approx }7\times 10^{-4}$) corrected event-by-event with the trigger and b tagging efficiency scale factors and $\mathcal {L}$ is the integrated luminosity. The fitted signal amounts to $3416\pm 79$ events. Taking into account the systematic uncertainties discussed in Sect. 6, the measured cross section is

2 $$\begin{aligned} \sigma _{{\mathrm{t}}\overline{{\mathrm{t}}}}=275.6\pm 6.1\,\text {(stat)} \pm 37.8\,\text {(syst)} \pm 7.2\,\text {(lumi)} ~\text {\,pb}. \end{aligned}$$

**Table 4:** Normalized differential ${\mathrm{t}}\overline{{\mathrm{t}}}$ cross section as a function of the $p_{\mathrm {T}}$ of the leading ($p_{\mathrm {T}} ^{(1)}$) and subleading ($p_{\mathrm {T}} ^{(2)}$) top quarks or antiquarks. The results are presented at particle level

$p_{\mathrm {T}}$ bin range ($\text {GeV}$ )	$\frac{1}{\sigma } {\mathrm{d}}\sigma /{\mathrm{d}}p_{\mathrm {T}} ^{(1)} (\text {GeV} ^{-1})$	Stat (%)	Exp. syst (%)	Theo. syst (%)
[0, 150]	$9.75\times 10^{-4}$	$\pm 5.2$	$-2.2, +1.5$	$-2.9, +1.0$
[150, 225]	$5.83\times 10^{-3}$	$\pm 4.4$	$-2.3, +2.3$	$-3.0, +1.2$
[225, 300]	$3.60\times 10^{-3}$	$\pm 5.0$	$-0.7, +1.3$	$-0.0, +3.1$
[300, 375]	$1.54\times 10^{-3}$	$\pm 6.8$	$-2.2, +2.5$	$-0.4, +4.3$
[375, 500]	$3.36\times 10^{-4}$	$\pm 14.6$	$-3.6, +1.8$	$-16.9, +7.4$

$p_{\mathrm {T}}$ bin range ($\text {GeV}$ )	$\frac{1}{\sigma } {\mathrm{d}}\sigma /{\mathrm{d}}p_{\mathrm {T}} ^{(2)} (\text {GeV} ^{-1})$	Stat (%)	Exp. syst (%)	Theo. syst (%)
[0, 150]	$2.33\times 10^{-3}$	$\pm 5.8$	$-2.5, +2.5$	$-3.3, +3.6$
[150, 225]	$5.46\times 10^{-3}$	$\pm 3.8$	$-1.5, +1.2$	$-3.4, +5.1$
[225, 300]	$2.66\times 10^{-3}$	$\pm 5.1$	$-1.4, +1.8$	$-3.9, +3.9$
[300, 375]	$7.67\times 10^{-4}$	$\pm 8.6$	$-1.7, +3.0$	$-4.0, +8.5$
[375, 500]	$1.80\times 10^{-4}$	$\pm 18.6$	$-5.0, +1.9$	$-11.4, +7.8$

The precision of the measured inclusive cross section is dominated by the systematic uncertainties, and in particular by those related to JES and b tagging.

In order to parametrize the dependence of the result on the top quark mass assumption, the measurement was repeated using signal simulated samples with different generated top quark masses (167.5 and 175.5 $\,\text {GeV}$). The choice of the generated mass affects both the extracted signal yield and the signal efficiency. The quadratic interpolation of the measurements with the three different top quark masses is

3 $$\begin{aligned} \frac{\sigma _{{\mathrm{t}}\overline{{\mathrm{t}}}}(m_{\mathrm{t}})}{\sigma _{{\mathrm{t}}\overline{{\mathrm{t}}}}(m_{\mathrm{t}}=172.5)}= & {} 1.0-2.4\times 10^{-2}(m_{\mathrm{t}}-172.5)\nonumber \\&+\,8.3\times 10^{-4}(m_{\mathrm{t}}-172.5)^2. \end{aligned}$$

### Differential cross sections

The size of the signal sample allows the differential measurement of the ${\mathrm{t}}\overline{{\mathrm{t}}}$ production cross section to be performed as a function of various observables. In order to confront the theoretical predictions, the differential cross sections are reported normalized to the inclusive cross section, resulting in a significant cancellation of systematic uncertainties.

The process of measuring the differential cross sections is identical to the inclusive case: in each bin of the observable used to divide the phase space, the signal is extracted from a template fit to the reconstructed top quark mass. Besides the physics interest, the choice of the observables used is mainly motivated by their correlation to $m_{{\mathrm{t}}}^\text {rec}$, and the ability to extract smooth signal and background templates. The variables chosen are the $p_{\mathrm {T}}$ of the two reconstructed top quarks. Figure 6 shows the fitted $m_{{\mathrm{t}}}^\text {rec}$ distributions in bins of the $p_{\mathrm {T}}$ of the leading top quark.

The differential measurements are first reported for the visible fiducial volume, as a function of the reconstructed top $p_{\mathrm {T}}$ (detector level), and then extrapolated to the parton and particle levels. The detector-level result is shown in Fig. 7 and is free of most of the systematic uncertainties affecting the inclusive measurement. The corresponding numerical values are reported in Table 2.

The parton-level results shown in Fig. 8 are obtained from the detector-level measurement, after correcting for bin migration effects and extrapolating to the full phase space using a bin-by-bin acceptance correction. The unfolding of the bin-migration effect is performed with the D’Agostini method [53], implemented in the RooUnfold package [54], using the migration matrix derived from the simulation. The uncertainty due to the modeling of the migration matrix and the phase-space extrapolation is estimated by repeating the unfolding and acceptance-correction procedures by varying the systematic sources described in Sect. 6. The numerical values of the normalized differential cross sections at parton level are reported in Table 3. It should be noted that there is a large extrapolation factor involved from the detector-level jets (7E–4 of the signal) to the full parton level, which results in large theoretical uncertainties.

In addition to the parton level, results are reported at particle level, in Fig. 9, in a phase space similar to the detector level by construction. This is defined as follows: first, particle jets are built in simulation from all stable particles (including neutrinos) with the same jet clustering algorithm as the detector jets. Then, starting from the six leading jets, the jets associated with B hadrons via matching in $\eta$–$\phi$ ($\Delta R < 0.25$) are identified as the b jet candidates. Events are further selected if $p_{\mathrm {T}} ^\text {4th jet} > 60~\,\text {GeV}$ and $p_{\mathrm {T}} ^\text {6th jet} > 40~\,\text {GeV}$ and if there are at least two b jets with $\Delta R_{{\mathrm{b}}{\mathrm{b}}} > 2.0$. For the selected events, a “pseudo top quark” is reconstructed from one b jet and the two closest non-b-tagged jets. The particle-level results are obtained in a similar way to the parton level, via unfolding and acceptance correction. The numerical values of the normalized differential cross sections at particle level are reported in Table 4.

The comparison of the measured and predicted differential top quark $p_{\mathrm {T}}$ shapes reveals that the models predict a harder spectrum, both in the leading and in the subleading top quark $p_{\mathrm {T}}$, in the phase space of the measurement. This effect is also reflected on the jet $p_{\mathrm {T}}$ distributions shown in Fig. 3. The powheg$+$herwig prediction is the closest to the data, but still shows a significant discrepancy. The parton-level results are accompanied by sizeable systematic uncertainties, dominated by the theoretical uncertainties due to the extrapolation to the full phase space. In contrast, the particle-level phase space is much closer to the visible one, and as a result the extrapolation uncertainties are smaller.

## Summary

A measurement of the ${\mathrm{t}}\overline{{\mathrm{t}}}$ production cross section has been performed in the all-jets final state, using pp collision data at $\sqrt{s}=8~\,\text {TeV}$ corresponding to an integrated luminosity of $18.4~{\,\text {fb}^{-1}}$. The measured inclusive cross section is $275.6\,\pm \, 6.1\,\text {(stat)} \pm 37.8\,\text {(syst)} \pm 7.2\,\text {(lumi)}$ $\text {\,pb}$ for a top quark mass of $172.5~\,\text {GeV}$, in agreement with the standard model prediction of $252.9^{+6.4}_{-8.6}\,(\text {scale})\pm 11.7\,(\text {PDF}+\alpha _S)$ $\text {\,pb}$ as calculated with the Top++ (v. 2.0) program [55] at next-to-next-to-leading order in perturbative QCD, including soft-gluon resummation at next-to-next-to-leading-log order [56], and assuming a top-quark mass $m_{{\mathrm{t}}}=172.5~\,\text {GeV}$. Also reported are the fiducial normalized differential cross sections as a function of the leading and subleading top quark $p_{\mathrm {T}}$. Compared to QCD predictions, the measurement shows a significantly softer top quark $p_{\mathrm {T}}$ spectrum. The differential cross sections are also extrapolated to the full partonic phase space, as well as to particle level, and can be used to tune Monte Carlo models.
